# Cyanobacteria as a Photosynthetic Chassis for Metabolic Pathway Engineering with Heterologous Gene Expression

**DOI:** 10.3390/cimb48060638

**Published:** 2026-06-19

**Authors:** Jessica Walshe, Sushanta Kumar Saha

**Affiliations:** Centre for Applied Bioscience Research, Technological University of the Shannon, Moylish Park, V94 E8Y Limerick, Ireland

**Keywords:** cyanobacteria, metabolic engineering, synthetic biology, heterologous metabolic pathways, genetic engineering, microbial cell factories, photosynthetic microorganisms

## Abstract

Cyanobacteria are increasingly recognised as photosynthetic chassis for sustainable metabolic engineering because oxygenic photosynthesis generates ATP and NADPH via the photosynthetic electron transport chain, which drive CO_2_ fixation through the Calvin–Benson–Bassham cycle into carbon intermediates that can be redirected toward engineered heterologous pathways. Their genetic tractability, CO_2_-fixing capacity, ecological adaptability, and relatively simple cellular organisation make them attractive platforms for developing low-carbon biotechnological processes. This review explores recent progress in engineering cyanobacteria for heterologous pathway construction, critically evaluating genetic tools including transformation methods, genome integration strategies, promoter systems, and CRISPR-based editing, with specific emphasis on challenges of direct relevance to phototrophic chassis: host–pathway metabolic compatibility, precursor supply, cofactor balancing between photosynthetic output and heterologous pathway demand, and achieving genetic stability in polyploid cyanobacterial genomes. The review also addresses key limitations with mechanistic context: metabolic burden from multi-gene pathway expression reduces growth rate and selects against producing cells; polyploidy delays complete chromosomal segregation of engineered constructs; slow photoautotrophic growth constrains volumetric productivity; native regulatory networks resist carbon flux redirection; and cultivation constraints—including light attenuation in dense cultures and mismatches between photosynthetic ATP/NADPH supply and heterologous pathway demand—further limit achievable yields.

## 1. Introduction

Cyanobacteria represent a diverse group of prokaryotic organisms characterised by their ability to perform oxygenic photosynthesis [1]. These microorganisms have played a fundamental role in the evolution and maintenance of Earth’s biosphere, primarily due to their capacity for oxygen-evolving photosynthesis and their ability to fix atmospheric carbon dioxide into organic biomass. This dual capability has profoundly influenced global biogeochemical cycles, making cyanobacteria essential contributors to ecosystem function [2].

As primary producers in both aquatic and terrestrial environments, cyanobacteria play a vital role in global carbon fixation and contribute substantially to the planet’s overall photosynthetic productivity [1,2]. Their metabolic pathways enable the conversion of inorganic carbon and solar energy into organic biomass, positioning them as key components of the global carbon cycle. Owing to their metabolic versatility and sustainability, cyanobacteria are increasingly being investigated as platforms for biotechnological applications. These applications include the development of microbial production systems capable of reducing reliance on fossil fuel–derived resources, thereby supporting the transition toward more sustainable energy and biomaterial production [3,4].

In recent years, there has been an increasing focus on the potential of photosynthetic microorganisms as microbial cell factories for the sustainable production of fuels, chemicals, and high-value metabolites. Traditional microbial hosts, such as *Escherichia coli* and *Saccharomyces cerevisiae*, have been extensively utilised in metabolic engineering due to their rapid growth rates, well-characterised genetic backgrounds, and sophisticated molecular toolkits [5,6]. However, these heterotrophic organisms rely on organic carbon substrates for their growth and the production of metabolites, which are typically derived from agricultural feedstocks or fossil-based resources. This reliance raises concerns regarding the overall sustainability of large-scale biotechnological production processes, as it may impose additional economic and environmental constraints [7].

In contrast, cyanobacteria possess the unique ability to grow photo-autotrophically by utilising light as their primary energy source while assimilating CO_2_ through the Calvin–Benson–Bassham cycle [1,8]. This metabolic capability enables cyanobacteria to convert solar energy and inorganic carbon directly into organic compounds, positioning them as candidates for sustainable biomanufacturing systems [8,9]. By integrating photosynthetic carbon fixation with engineered metabolic pathways, these microorganisms can function as light-driven microbial factories, producing valuable chemicals directly from CO_2_ [9,10]. As a result, cyanobacteria are increasingly being explored as alternative hosts for metabolic engineering and synthetic biology applications aimed at developing carbon-neutral or even carbon-negative production platforms [10,11]. Realising this biosynthetic potential requires overcoming several inherent biological constraints—including slow photoautotrophic growth, polyploid genome architecture, sensitivity of photosynthetic metabolism to redox and light fluctuations, and the metabolic burden imposed by heterologous pathway expression—each of which is examined in detail in Section 4 and Section 5 of this review.

However, whether cyanobacterial bioproduction achieves carbon-neutral or carbon-negative outcomes in practice is not an inherent property of the platform but depends critically on several connected factors. Life-cycle analyses of photosynthetic cyanobacterial ethanol production have reported a 67–87% reduction in greenhouse gas emissions relative to gasoline under optimised conditions [12]; however, the carbon balance is sensitive to the source of CO_2_ supplied, with dedicated life-cycle assessment of algal biofuel production demonstrating that the choice between waste industrial flue gas, biogenic CO_2_, and commercially supplied fossil-derived CO_2_ produces substantially different greenhouse gas outcomes. This use of fossil-derived CO_2_ can significantly negate the carbon benefit [13]. Energy inputs for cultivation, mixing, and downstream product purification represent a further critical determinant: large-scale life-cycle modelling of cyanobacterial biobutanol production found that the electricity demand for these processes fully offsets the photosynthetic carbon benefit when conventional grid electricity is used, and that even with renewable electricity the resulting greenhouse gas balance was only marginally more favourable than fossil reference values [14]. The same modelling demonstrated that greenhouse gas emissions are most sensitive to organism-level product yield and volumetric productivity—parameters at which current engineered cyanobacterial systems, operating at milligram-per-litre-per-day scale, fall substantially short of the thresholds assumed in favourable life-cycle scenarios [15]. Furthermore, carbon-neutral or carbon-negative bioproduction from cyanobacteria has not been demonstrated at industrial cultivation scale in any published study to date, and realising this potential will require simultaneous advances in product yield, productivity, renewable energy integration, and CO_2_ sourcing strategy.

Recent advancements in synthetic biology and metabolic engineering have greatly broadened the potential of cyanobacteria as platforms for heterologous biosynthesis [10,16]. Over the past decade, significant progress has been achieved in the development of genetic tools for cyanobacterial metabolic engineering, including improved transformation methods, modular DNA assembly systems, synthetic promoters, and CRISPR-based genome editing technologies [10,16,17]. These developments have enabled the efficient introduction and expression of heterologous metabolic pathways within cyanobacterial hosts, supporting the biosynthesis of compounds that are not naturally produced by these organisms [10,18]. As a result, cyanobacteria are increasingly being investigated as photosynthetic platforms to produce a diverse range of metabolites, including alcohols, hydrocarbons, fatty acids, terpenoids, and other value-added molecules [18,19].

Several cyanobacterial species have become established as model organisms for research in metabolic engineering and synthetic biology. Most extensively studied unicellular cyanobacterial strains are *Synechocystis* sp. PCC 6803, *Synechococcus elongatus* PCC 7942, *Synechococcus* sp. PCC 7002, and the most studied filamentous cyanobacterium is *Anabaena* sp. PCC 7120 [20]. These organisms exhibit distinct differences in physiological characteristics, growth behaviour, and genetic tractability; however, together they provide versatile experimental platforms for investigating photosynthetic metabolism and developing engineered biosynthetic pathways. For example, *Synechocystis* sp. PCC 6803 is widely utilised due to its natural competence and high amenability to genetic manipulation, whereas *Synechococcus* species are often preferred for their relatively rapid growth rates and well-characterised physiology [21,22]. The expanding availability of cyanobacterial genomic resources has greatly facilitated genetic and systems-level studies. As of May 2026, public databases such as NCBI list approximately 9000 cyanobacterial genome assemblies [23], while curated analyses indicate that several hundred of these, around 284 genomes, are available at complete-genome or chromosome-level assembly quality [24]. Additionally, several strains display natural competence or can be efficiently transformed using techniques such as conjugation or electroporation, enabling targeted genome modification through homologous recombination and related genetic engineering strategies [25].

Engineering cyanobacteria especially for heterologous biosynthesis typically involves introducing multi-gene metabolic pathways sourced from non-native organisms, often necessitating the coordinated expression of multiple enzymatic steps [26]. To be functional, these pathways need to redirect native metabolic intermediates toward the synthesis of target compounds through the regulated activity of sequential enzymes. Through metabolic pathway engineering, model cyanobacterial strains can be redirected to produce non-native or enhanced levels of target compounds by channelling photosynthetically fixed carbon into different biosynthetic routes. Although these products generally share CO_2_ as the common upstream carbon source, their immediate precursors and pathways differ: ethanol and isobutanol are mainly derived from pyruvate-based pathways [27], fatty acids and alkanes from acetyl-CoA/fatty acyl-ACP metabolism, including the AAR/ADO alkane pathway [28] and terpenoids from the MEP pathway, which uses pyruvate and glyceraldehyde-3-phosphate as precursors [29]. Collectively, these examples highlight the potential of cyanobacteria as light-driven microbial platforms for converting CO_2_ and solar energy into diverse biofuels and value-added molecules.

However, despite recent progress, employing cyanobacteria as hosts for heterologous metabolic pathway engineering still poses several biological and technical challenges. Compared with traditional heterotrophic microbial hosts, cyanobacteria generally exhibit slower growth rates and lower volumetric productivity, which may limit their suitability for industrial applications [30]. Additionally, many cyanobacterial species have polyploid genomes, meaning that multiple copies of the chromosome are present within a single cell. For example, the filamentous cyanobacterium *Anabaena* sp. PCC 7120 has been reported to contain approximately eight chromosome copies per wild-type cell, and its complete genome comprises a 6,413,771 bp chromosome together with six plasmids [31]. This genomic configuration complicates genetic manipulation and often delays the segregation of engineered mutations or inserted genetic constructs after genome editing. Moreover, the efficiency of heterologous metabolite production can be limited by various physiological factors, including constraints on regulatory barriers within native metabolic networks, the metabolic burden imposed by multi-gene pathway expression, and the energetic demands associated with photosynthetic processes and potential limitations in photosynthetic efficiency under laboratory or industrial cultivation conditions [19,32].

In addition to these constraints, another challenge in engineering cyanobacteria for heterologous biosynthesis is the metabolic burden caused by expressing foreign genes and complex biosynthetic pathways [3]. Introducing heterologous enzymes can cause physiological stress to cyanobacterial cells and divert resources away from growth and native metabolic functions [16]. Additionally, differences in codon usage, protein-folding needs, and cofactor availability may affect the stability and efficiency of heterologous enzyme expression in cyanobacterial hosts. These factors can collectively reduce pathway efficiency and restrict product yields [33]. These limitations collectively define the central challenges that this review seeks to address within a structured framework. The complications arising from polyploidy—including delayed chromosomal segregation following genome editing and the persistence of mixed genotypes—directly motivate the critical evaluation of CRISPR-based editing tools, homologous recombination strategies, and segregation-accelerating approaches presented in Section 3. The metabolic burden imposed by multi-gene pathway expression, together with the challenges of codon usage incompatibility, heterologous protein folding, and cofactor imbalance between photosynthetic supply and heterologous pathway demand, underpin the pathway design strategies, precursor engineering approaches, and cofactor balancing considerations examined in Section 4. The constraints of slow photoautotrophic growth and low volumetric productivity relative to heterotrophic hosts, alongside the cultivation and scale-up limitations discussed in Section 5, frame the broader question of how cyanobacterial metabolic engineering can progress from laboratory demonstration toward industrially relevant performance. By structuring the review around these specific challenges rather than treating them as peripheral observations, the present work aims to provide a more mechanistically grounded assessment of the current state and future trajectory of cyanobacterial pathway engineering.

Recent research has therefore focused on expanding the synthetic biology toolbox available for cyanobacterial engineering while improving strategies for metabolic pathway design and optimisation [10]. Advances in this area include the development of synthetic promoter libraries, optimised ribosome binding sites, modular DNA assembly platforms, and CRISPR-based genome engineering tools, all of which have significantly improved the ability to regulate gene expression and manipulate metabolic networks in cyanobacterial systems [16,17]. In parallel, systems biology approaches—including transcriptomics, proteomics, and metabolic modelling—are increasingly employed to identify metabolic bottlenecks and guide rational strain-engineering strategies aimed at improving pathway efficiency, metabolic balance, and overall target product yields [34].

Despite growing interest in cyanobacteria as metabolic engineering platforms, existing literature has largely catalogued individual genetic tools and pathway examples without providing integrated analysis of the specific design principles, host–pathway compatibility challenges, cofactor requirements, and performance determinants that govern whether heterologous pathways succeed in phototrophic chassis—a gap that this review specifically addresses. However, the effective application of cyanobacterial cell factories requires a comprehensive understanding of the genetic tools available for strain engineering, the strategies used to construct heterologous metabolic pathways, and the molecular limitations that currently constrain their productivity [15]. This review examines recent advances in engineering of cyanobacteria as chassis organisms for heterologous metabolic pathway integration. It outlines the biological characteristics that make cyanobacteria attractive hosts for synthetic biology applications, provides an overview of the genetic tools and transformation strategies used for their manipulation, and discusses recent progress in constructing heterologous metabolic pathways in cyanobacterial hosts. Finally, the major molecular and physiological challenges that continue to limit cyanobacterial metabolic engineering are highlighted, together with emerging strategies aimed at overcoming these limitations.

## 2. Cyanobacteria as Photosynthetic Chassis for Metabolic Engineering

### 2.1. Overview of Cyanobacterial Biology

Cyanobacteria are a morphologically and physiologically diverse group of prokaryotic microorganisms distinguished by their ability to perform oxygenic photosynthesis [35]. They are widely regarded as ancient organisms of major evolutionary significance, with their emergence closely linked to the evolution of oxygenic photosynthesis and the subsequent rise in atmospheric oxygen during the Great Oxidation Event approximately 2.6 billion years ago. Through the oxidation of water and the release of molecular oxygen as a by-product of photosynthetic activity, cyanobacteria profoundly altered the chemistry of Earth’s atmosphere and oceans, ultimately enabling the evolution of aerobic metabolism and more complex life forms [36].

A key feature of cyanobacteria is the presence of an internal thylakoid membrane system that contains the protein-pigment complexes responsible for the light reactions of photosynthesis, including photosystem II and photosystem I. These photosystems work in sequence to absorb light energy, drive photosynthetic electron transport [PET], and produce ATP and NADPH, which are then utilised for carbon fixation via the Calvin–Benson–Bassham cycle [37]. This unified photosynthetic apparatus allows cyanobacteria to grow as photoautotrophs, using light as an energy source and CO_2_ as their main carbon. As a result, cyanobacteria have attracted significant interest both as model organisms for studying photosynthesis and as potential hosts for sustainable biotechnological and metabolic pathway engineering pursuits [38].

Cyanobacteria inhabit an exceptionally broad range of ecological niches, including freshwater, marine, terrestrial, and extreme environments such as hot springs, deserts, and polar habitats [39]. They appear in a variety of morphological forms, ranging from unicellular and colonial species to filamentous organisms capable of cellular differentiation. In certain filamentous cyanobacteria, such as *Anabaena* species, specialised cells known as heterocysts develop under nitrogen-limiting conditions and facilitate biological nitrogen fixation [40].

Ecologically, cyanobacteria serve as major primary producers and significantly contribute to the global carbon [C] and nitrogen [N] cycling [1]. In aquatic ecosystems, they play vital roles in biomass production and nutrient turnover. At the same time, in terrestrial environments, they assist in soil formation, the development of biological soil crusts, and the maintenance of ecosystems. Their evolutionary longevity, ecological adaptability, and distinctive metabolic abilities have established cyanobacteria as important model organisms in microbiology and increasingly appealing hosts for genetic and metabolic pathway engineering [3].

### 2.2. Metabolic and Physiological Features Relevant to Biotechnology

The metabolic and physiological characteristics of cyanobacteria make them particularly attractive organisms for various biotechnological applications. Central to cyanobacterial metabolism is the PET chain, which functions within the thylakoid membranes and produces both ATP and reducing power in the form of NADPH during the light-dependent reactions of photosynthesis. These energy carriers supply the chemical energy necessary for carbon fixation and a variety of biosynthetic processes within the cell [41]. In cyanobacteria, the fixation of inorganic carbon is further supported by specialised protein microcompartments known as carboxysomes, which encapsulate key enzymes involved in carbon assimilation, most notably ribulose-1,5-bisphosphate carboxylase/oxygenase [RuBisCO] [42]. Carboxysomes serve as part of the cyanobacterial carbon-concentrating mechanism, enhancing the efficiency of carbon fixation by increasing the local concentration of CO_2_ around RuBisCO and thereby reducing oxygenation reactions that cause photorespiration. The presence of this carbon-concentrating system enhances the utilisation of inorganic carbon and improves the overall metabolic efficiency of cyanobacteria, thus strengthening their potential as platforms for photosynthetically driven biomanufacturing [42].

Another important physiological trait observed in many cyanobacterial species is their capacity to fix atmospheric nitrogen. In filamentous cyanobacteria such as *Anabaena* sp., nitrogen fixation takes place within specialised cells called heterocysts. These heterocysts differentiate from vegetative cells when nitrogen is scarce and create a microoxic environment that facilitates the activity of the oxygen-sensitive enzyme nitrogenase [43]. These heterocysts possess structural and metabolic adaptations that restrict oxygen diffusion while supporting the high-energy requirements of nitrogen fixation. Although nitrogen fixation is not a universal trait among cyanobacterial strains commonly used in metabolic engineering, this metabolic capability demonstrates the physiological versatility of cyanobacteria and their ability to adapt to nutrient-limited environments [44]. Such flexibility contributes to the ecological success of cyanobacteria and highlights their capacity to grow under a range of environmental circumstances [43].

Cyanobacteria have relatively simple nutritional requirements compared to many heterotrophic microbial hosts. Their growth can be maintained using light as an energy source in conjunction with carbon dioxide, water, and inorganic nutrients, thereby eliminating the need for organic carbon substrates typically necessary for organisms such as *Escherichia coli* or yeast [15]. This metabolic capability holds significant implications for the development of sustainable production systems, as it allows for the direct conversion of CO_2_ and solar energy into biomass and desired compounds [45]. As a result, cyanobacteria represent a candidate platform for integrating microbial biomanufacturing with carbon capture technologies and renewable energy sources, promoting more sustainable and resource-efficient biotechnological processes [3].

Alongside their metabolic versatility, many cyanobacterial species demonstrate significant environmental resilience. Several taxa can tolerate variations in light intensity, temperature, and nutrient levels, reflecting their long evolutionary history of adaptation to diverse and often changing natural environments [46]. This physiological robustness is especially beneficial for biotechnological uses, particularly in large-scale or outdoor cultivation systems where environmental conditions cannot always be precisely managed. In these systems, organisms must sustain stable metabolic activity despite fluctuations in light, temperature, and nutrient supply. Additionally, many cyanobacteria feature regulatory mechanisms that enable them to modulate their photosynthetic activity and metabolic flux in response to varying environmental conditions. These adaptive strategies contribute to cellular stability and support sustained growth across diverse environmental contexts [47]. From a biotechnological perspective, this physiological flexibility can support the development of scalable production platforms, wherein cyanobacterial cultures are cultivated under non-sterile or semi-controlled cultivation conditions [15]. Translating laboratory engineering to industrial practice requires selection between closed photobioreactors—tubular, flat-panel, or airlift designs offering contamination control and precise regulation of light, pH, CO_2_, and temperature—and open raceway ponds, which are cheaper to construct and operate but expose cultures to contamination and seasonal productivity variation. Industrial-scale raceway cultivation of *Arthrospira platensis* at 300 m^2^ under natural sunlight has demonstrated year-round operation, though seasonal light variation caused up to 65% loss of potential biomass productivity and harvesting inefficiencies a further 30% loss [48]. At pilot scale, a cyanobacterial consortium maintained in a 1150 L outdoor tubular PBR for 130 consecutive days at pH 10.4–11.4 achieved areal productivities of 3.1–5.8 g m^−2^ d^−1^ while enabling direct atmospheric CO_2_ capture, demonstrating that high-pH alkaliphilic cultivation supports stable long-term outdoor production [49]. For genetically engineered strains, additional challenges include maintaining genetic stability under natural light-dark cycles and the selective pressures of semi-continuous operation.

Contextualising cyanobacteria against the most widely used heterotrophic hosts highlights both their limitations and unique value. *E. coli* offers a doubling time of approximately 20 min, the most mature synthetic biology toolkit of any organism, and volumetric productivities at the grams per litre per hour scale from glucose feedstocks. *S. cerevisiae* provides superior product and solvent tolerance, a well-developed toolkit for complex natural products, and established industrial infrastructure, though both hosts require processed organic carbon feedstocks costing approximately €0.25–0.45 per kg and carry upstream agricultural carbon costs [3]. Cyanobacteria grow more slowly (doubling times of 1.5–10 h) with a less mature toolkit, but uniquely fix CO_2_ directly using solar energy, eliminating organic carbon feedstock requirements entirely and offering net carbon-neutral or carbon-negative production potential that neither heterotrophic host can match [15].

Together, the combination of photoautotrophic metabolism, efficient carbon assimilation, relatively simple nutritional needs, and environmental resilience has contributed to growing interest in cyanobacteria as platforms for sustainable biotechnological applications. These traits support the advancement of photosynthetically driven systems capable of converting solar energy and CO_2_ into a variety of valuable chemical products, such as fuels, polymers, and high-value metabolites.

### 2.3. Model Cyanobacterial Strains Used for Genetic Engineering

While cyanobacteria represent a diverse phylum with thousands of species exhibiting a broad spectrum of physiological and ecological traits, only a limited number have been extensively utilised as model organisms in genetic engineering and synthetic biology research. The choice of suitable cyanobacterial hosts for metabolic engineering primarily depends on factors such as growth rate, genetic manipulability, availability of molecular tools, and the level of understanding of their physiology and genome [50]. Over recent decades, a small subset of cyanobacterial species has emerged as principal experimental platforms for studying photosynthetic metabolism and establishing engineered biosynthetic pathways [51]. Among the most extensively employed cyanobacterial model organisms are the freshwater strains *Synechocystis* sp. PCC 6803, *Synechococcus elongatus* PCC 7942, the filamentous nitrogen-fixing cyanobacterium *Anabaena* sp. PCC 7120, along with *Synechococcus* sp. PCC 7002, a marine strain capable of growth across a broad salinity range [52]. These organisms exhibit significant variation in their physiological properties, metabolic capabilities, and environmental adaptations; nonetheless, each provides unique advantages for genetic manipulation and metabolic engineering endeavours. Collectively, these model strains have established foundational platforms for cyanobacterial genetic tool development and have facilitated notable progress in photosynthetic biotechnology and synthetic biology research [3,10].

#### 2.3.1. *Synechocystis* sp. PCC 6803

Among cyanobacterial model organisms, *Synechocystis* sp. PCC 6803 stands out as one of the most extensively studied and widely utilised strains in the fields of photosynthetic biotechnology and metabolic engineering [53]. This unicellular freshwater cyanobacterium is particularly valued for its innate competence for DNA uptake, facilitating the introduction of exogenous DNA into its genome via homologous recombination [54]. Consequently, *Synechocystis* sp. PCC 6803 has established itself as a key model cyanobacterium for examining photosynthesis, carbon metabolism, and the genetic regulation underlying cyanobacterial physiology [55]. The complete genome sequence of *Synechocystis* sp. PCC 6803 was among the first cyanobacterial genomes to be fully characterised, providing a crucial foundation for systems biology and genetic manipulation studies [56]. The extensive genomic data have supported the development of a variety of genetic tools, such as promoter libraries, plasmid vectors, and genome-editing techniques, which enable targeted modifications of metabolic pathways [10]. Additionally, the well-characterised metabolic network of this organism has made it a preferred host for introducing and optimising heterologous biosynthetic pathways in cyanobacterial metabolic engineering [57]. In addition to its genetic tractability, *Synechocystis* sp. PCC 6803 exhibits notable physiological flexibility, capable of growing photoautotrophically and mixotrophically under laboratory conditions [58]. This metabolic diversity has enabled comprehensive studies of carbon metabolism and cellular energy balance, making the strain particularly valuable for investigating strategies to optimise metabolic fluxes in engineered pathways [3]. Consequently, *Synechocystis* sp. PCC 6803 remains a central organism for the development and assessment of genetic engineering techniques in cyanobacteria.

#### 2.3.2. *Synechococcus elongatus* PCC 7942

Another commonly used cyanobacterial model organism is *Synechococcus elongatus* PCC 7942, a unicellular freshwater cyanobacterium that has been extensively investigated in both photosynthesis research and synthetic biology. This strain has become a key experimental platform due to its relatively fast growth, well-characterised physiology, and the availability of established genetic manipulation techniques [59,60]. Like *Synechocystis* sp. PCC 6803, *Synechococcus elongatus* PCC 7942 is naturally competent and can undergo natural transformation, whereby extracellular DNA is taken up directly from the surrounding medium. In this strain, DNA can be introduced as replicative plasmids, integrative plasmids, or linear PCR fragments; when the introduced DNA contains regions homologous to the chromosome, it can integrate into defined neutral sites through homologous recombination, enabling stable gene insertion, deletion, or replacement. Conjugation from *E. coli* provides an additional route for delivering transferable or broad-host-range plasmids, while electroporation has been reported for cyanobacteria but is less routinely used for *S. elongatus* PCC 7942 [61]. One of the most significant features of *S. elongatus* PCC 7942 is its well-characterised circadian clock system, which regulates a variety of physiological processes, including photosynthesis, metabolism, and cell division. Investigations into circadian rhythms in this organism have yielded valuable insights into the temporal regulation of metabolic activity in cyanobacteria. From a biotechnological standpoint, this endogenous circadian regulation has also garnered interest in the metabolic engineering, as it can affect the timing and efficiency of engineered metabolic pathways [62]. The streamlined genome and predictable genetic behaviour of *S. elongatus* PCC 7942 have facilitated the development of various synthetic biology tools, such as inducible promoters, modular genetic circuits, and genome integration systems. As a result, this organism has become a widely utilised platform for constructing and assessing engineered metabolic pathways in cyanobacteria. Its combination of genetic tractability, well-characterised physiology, and relatively rapid growth rate positions *S. elongatus* PCC 7942 as a significant model for advancing cyanobacterial metabolic engineering strategies [63].

#### 2.3.3. *Synechococcus* sp. PCC 7002

Another cyanobacterial strain that has gained considerable attention in recent years is *Synechococcus* sp. PCC 7002. This unicellular cyanobacterium was initially isolated from a marine environment and is distinguished by its capacity to tolerate high salinity levels and elevated light intensities [64,65]. The rapid growth, high-light tolerance, and broad salinity tolerance of *Synechococcus* sp. PCC 7002 distinguish it from many freshwater cyanobacterial models. These traits allow cultivation under saline and variable outdoor conditions, support higher biomass productivity, and may reduce contamination by freshwater microorganisms that are less able to tolerate elevated salinity. A significant advantage of *Synechococcus* sp. PCC 7002 is its comparatively rapid growth rate compared with other cyanobacteria used in metabolic engineering. Under optimal laboratory conditions, this strain can attain higher biomass productivity than several commonly used cyanobacterial hosts, thereby enhancing its potential for industrial biotechnology applications [64,66]. Besides its favourable growth properties, *Synechococcus* sp. PCC 7002 has a well-characterised genome and has demonstrated amenability to genetic modification via homologous recombination-based techniques [64]. Its genome comprises one chromosome of approximately 3.0 Mb and six plasmids—pAQ1, pAQ3, pAQ4, pAQ5, pAQ6 and pAQ7—with plasmid sizes of approximately 4.8, 16, 32, 39, 124 and 186 kb, respectively [67].

The physiological resilience of *Synechococcus* sp. PCC 7002, including its ability to withstand diverse environmental conditions and high light intensities, positions it as a candidate for the development of photosynthetic biotechnological systems [68]. This strain has been reported to grow rapidly under light intensities of up to 2000 µmol photons m^−2^ s^−1^, while also being reported to survive under extremely high irradiance, up to approximately 4500 µmol photons m^−2^ s^−1^, equivalent to about two times peak sunlight [69]. Its marine origin allows cultivation in saline media, potentially reducing competition for freshwater resources and facilitating the scaling of phototrophic bioprocesses. As a result, this organism is increasingly studied as a viable platform for cyanobacterial metabolic engineering and synthetic biology research.

#### 2.3.4. *Anabaena* sp. PCC 7120

The filamentous cyanobacterium *Anabaena* sp. PCC 7120 serves as a significant model organism within cyanobacterial research, particularly in developmental biology, genetics and physiology [70]. Unlike the previously mentioned unicellular strains, *Anabaena* sp. PCC 7120 develops multicellular filaments made up of chains of vegetative cells. These cells can undergo differentiation into specialised cell types when exposed to specific environmental stimuli. A prominent example of such cellular differentiation is the development of heterocysts, which are specialised cells that facilitate atmospheric nitrogen [N2] fixation under nitrogen limitation conditions. Heterocysts create a microoxic environment that shields the oxygen-sensitive enzyme nitrogenase, thereby enabling N2-fixation to proceed concurrently with oxygenic photosynthesis in adjacent vegetative cells [71]. The genome of *Anabaena* sp. PCC 7120 has been entirely sequenced, demonstrating a relatively large and intricate genetic composition in comparison to many unicellular cyanobacteria [72]. This genetic complexity underpins the organism’s ability for cellular differentiation and the coordination of metabolic activities across multicellular filaments. Over the last twenty years, *Anabaena* sp. PCC 7120 has emerged as a significant model system for studying cell differentiation, intercellular communication, and nitrogen metabolism in cyanobacteria. Notably, the exchange of metabolites between vegetative cells and heterocysts offers a well-characterised framework for exploring coordinated metabolic processes within multicellular microbial communities [71,73]. From a biotechnology standpoint, the multicellular structure of *Anabaena* presents distinct opportunities for metabolic engineering. The presence of distinct cell types facilitates spatial separation of metabolic processes within the filament, which can be beneficial for expressing complex or oxygen-sensitive pathways [74]. Additionally, *Anabaena* sp. PCC 7120 can be genetically engineered through conjugation-based techniques, allowing the insertion of heterologous genes and designed metabolic pathways. These features have contributed to growing interest in using *Anabaena* sp. PCC 7120 as a chassis organism for synthetic biology and metabolic pathway engineering research [33,75].

#### 2.3.5. *Synechococcus* sp. PCC 11901

*Synechococcus* sp. PCC 11901 is a fast-growing marine cyanobacterium that has emerged as a high-priority non-model chassis due to its capacity for sustained biomass accumulation to very high cell densities of up to 30 g dry cell weight L^−1^—comparable to fed-batch *E. coli*—under photoautotrophic conditions. The strain tolerates high light intensities exceeding 900 µmol photons m^−2^ s^−1^, temperatures up to 43 °C, and salinities more than twice that of seawater, making it well suited to outdoor and non-sterile cultivation scenarios [76]. Despite these favourable physiological properties, genetic tools for engineering PCC 11901 were historically limited. Victoria et al. addressed this directly by developing a comprehensive engineering toolbox based on the CyanoGate Modular Cloning system, characterising neutral genomic integration sites, testing a suite of constitutive and inducible promoters, and demonstrating a novel 2,4-diacetylphloroglucinol-inducible PhlF repressor system with a 228-fold dynamic range—the first demonstration of this system in any cyanobacterium [76]. The same study established conditional CRISPRi-based gene knockdown and a CRISPR-Cas12a markerless genome editing approach achieving single-insertion efficiencies of 31–81%. These tools have since been extended further, with 14 additional neutral genomic sites characterised, promoter libraries providing an approximately 800-fold expression range, and a cobalamin-independent chassis strain constructed to reduce cultivation costs [77]. Product demonstrations including free fatty acid titres of up to 1.5 g/L—comparable to similarly engineered heterotrophic organisms—and astaxanthin biosynthesis illustrate the productive potential of this strain when paired with an expanding genetic toolkit. A genome-scale metabolic model has also been reconstructed for PCC 11901 to guide rational engineering decisions through flux balance analysis [78].

## 3. Genetic Tools for Cyanobacterial Engineering

The effective use of cyanobacteria as platforms for metabolic engineering and synthetic biology largely depends on the availability of reliable genetic tools that facilitate precise manipulation of their genomes and metabolic pathways [79]. Over recent decades, significant progress has been achieved in developing molecular techniques that enable the introduction, modification, and regulation of genetic elements within cyanobacterial cells. Such tools allow researchers to construct heterologous metabolic pathways, alternative metabolic networks, and regulate gene expression to optimise metabolic flux within engineered systems [50]. Early efforts in cyanobacterial genetic engineering primarily relied on homologous recombination-based approaches for integrating foreign DNA into the genome. Although these methods remain prevalent, advancements in synthetic biology have considerably broadened the range of available genetic tools. Contemporary strategies incorporate modular DNA assembly techniques, synthetic regulatory elements, and genome editing technologies that enable more precise and efficient manipulation of cellular metabolism. These developments have significantly advanced the capacity to design and implement engineered metabolic pathways in cyanobacterial hosts [33].

Despite these advances, genetic manipulation in cyanobacteria can still present unique challenges compared to traditional heterotrophic microbial hosts. Many cyanobacterial species possess polyploid genomes with multiple chromosome copies, complicating genome editing and delaying the segregation of engineered mutations. Additionally, differences in transformation efficiency, promoter activity, and gene regulatory mechanisms among species often necessitate strain-specific optimisation of genetic strategies. As a result, ongoing development and refinement of genetic tools remain crucial for enhancing the efficiency and reliability of cyanobacterial metabolic engineering.

### 3.1. DNA Introduction Methods in Cyanobacteria

The introduction of exogenous DNA into cyanobacterial cells is a critical step in the fields of genetic engineering and synthetic biology. Various transformation techniques have been developed to facilitate the delivery of plasmid DNA or linear DNA constructs into cyanobacterial hosts [79]. The efficiency of these techniques varies across different species and strains, primarily due to differences in cell envelope architecture, physiological traits, and natural competence mechanisms. The most frequently employed methods for cyanobacterial transformation include natural competence, conjugation-based DNA transfer, and electroporation [3].

Natural competence refers to the ability of certain cyanobacteria to actively uptake extracellular DNA from their surrounding environment. Some cyanobacteria, notably *Synechocystis* sp. PCC 6803, demonstrates natural competence and can incorporate exogenous DNA directly into their genomes via homologous recombination [79,80]. This characteristic greatly simplifies genetic manipulation, as DNA fragments with regions homologous to the target genomic locus can be introduced without specialised delivery systems. Once internalised, the DNA integrates into the chromosome through recombination mechanisms, allowing for targeted gene disruption, replacement, or insertion.

For cyanobacterial species lacking natural competence, conjugation-based DNA transfer is widely used. In this approach, plasmid DNA is mobilised from a donor bacterium, typically *Escherichia coli* [e.g., DH10B or DH5α carrying pRL623 and pRL443 plasmids], into cyanobacterial recipient cells via biparental conjugation [81]. Conjugation has been successfully utilised across various cyanobacterial strains, including *Anabaena* sp. PCC 7120 and species of *Synechococcus*, and continues to be one of the most dependable techniques for introducing foreign DNA into filamentous or genetically less amenable cyanobacteria. This approach is particularly advantageous for transferring large plasmids or complex genetic constructs that are challenging to introduce via other transformation methods [82].

Electroporation is a frequently utilised method for the transformation of cyanobacteria. This technique employs short, high-voltage electrical pulses to transiently disrupt the cell membrane, facilitating the entry of DNA molecules into the cell [83]. While electroporation is common in heterotrophic bacteria such as *Escherichia coli*, its application in cyanobacteria presents specific challenges. Many cyanobacterial species possess relatively robust cell envelopes, including comparatively thick peptidoglycan layers and extracellular polysaccharide sheaths, which can hinder DNA uptake and reduce transformation efficiency. In addition, successful electroporation in cyanobacteria is often strain- and protocol-dependent, as electrical treatment conditions must be carefully optimised to preserve cell viability while enabling DNA entry [3,84]. The presence of restriction–modification systems in certain strains may also lead to degradation of foreign DNA, further limiting transformation success. Consequently, effective electroporation in cyanobacteria often necessitates meticulous optimisation of growth conditions, DNA preparation, and electrical pulse parameters. Despite these obstacles, electroporation remains an important technique for introducing plasmid DNA into specific cyanobacterial strains, where alternative methods may be less effective [85,86].

Collectively, these DNA delivery strategies form the foundation of genetic manipulation in cyanobacteria and facilitate the introduction of engineered constructs necessary for metabolic pathway engineering. The choice of transformation method often depends on the specific cyanobacterial strain, the size and type of genetic construct, and the intended genomic integration approach [3].

### 3.2. Vectors and Genome Integration Strategies

Following the introduction of exogenous DNA into cyanobacterial cells, it is essential to achieve stable maintenance and expression of the introduced genetic material to facilitate reliable genetic engineering [87]. In cyanobacteria, this goal is typically accomplished either through plasmid-based expression systems or by integrating foreign DNA directly into the host genome via single or double homologous recombination. The selection between these methods depends on several factors, including the required stability of gene expression, the size of the introduced construct, and the specific physiological and genetic characteristics of the cyanobacterial strain [88]. Plasmid vectors are frequently employed for the expression of heterologous genes in cyanobacteria. These vectors can either replicate independently within the host cell or function as delivery systems for chromosomal integration. Replicative plasmids generally contain origins of replication that facilitate autonomous maintenance within the cyanobacterial host [89]. Nevertheless, plasmid stability can differ significantly among various strains, and plasmid loss may occur during extended cultivation in the absence of selective antibiotics pressure. Consequently, many metabolic engineering approaches prefer chromosomal integration to ensure the stable inheritance of engineered genetic constructs.

In cyanobacterial genetic engineering, plasmid systems are typically classified as either shuttle plasmids or suicide plasmids, depending on their specific applications. Shuttle plasmids can replicate in multiple host organisms, usually *Escherichia coli* and the target cyanobacterial strain, which simplifies plasmid construction and amplification in *E. coli* prior to transfer into cyanobacteria [88,90]. These plasmids contain compatible origins of replication that ensure maintenance in both hosts and are commonly employed for transient gene expression or for introducing genetic constructs into cyanobacterial cells [90]. Conversely, suicide plasmids lack a functional origin of replication in cyanobacterial hosts and therefore cannot replicate independently once introduced into the cyanobacterial cell. Instead, they are engineered to integrate into the chromosome through homologous recombination, facilitating stable insertion or replacement of genomic sequences. As such, suicide plasmids are widely used in targeted genome modification and gene knockout techniques within cyanobacterial genetic engineering [91,92].

Chromosomal integration in cyanobacteria is predominantly accomplished via homologous recombination. This mechanism involves the exchange of DNA segments containing sequences homologous to specific genomic loci, thereby facilitating targeted insertion, deletion, or replacement of genetic material [51]. Homologous recombination remains one of the most recognised and reliable methods for stable genetic modification in cyanobacterial research. To minimise disruption of essential cellular functions, engineered constructs are typically inserted into designated neutral genomic sites. These loci represent regions of the genome that can accommodate foreign DNA without notably impacting host growth or metabolic activity [93]. Neutral integration sites have been identified and characterised in commonly used cyanobacterial model organisms, such as *Synechocystis* sp. PCC 6803 and *Synechococcus elongatus* PCC 7942, providing reliable genomic locations for the stable insertion of heterologous genes and engineered metabolic pathways [94,95].

Together, plasmid-based expression systems and chromosomal integration strategies constitute the core methodologies in genetic pathway engineering of cyanobacteria. These techniques facilitate the stable introduction and expression of heterologous genes, which are critical for constructing engineered metabolic pathways and developing cyanobacterial strains for applications in synthetic biology and metabolic engineering [96].

### 3.3. Promoters and Gene Expression Systems in Cyanobacteria

Precise regulation of gene expression is essential for metabolic engineering in cyanobacteria. When heterologous metabolic pathways are introduced into a host, fine-tuning the expression levels of individual enzymes is often necessary to optimise metabolic flux and avoid the buildup of toxic intermediates. For this reason, the development of robust gene expression systems has become a key area of focus in cyanobacterial synthetic biology research [79].

Promoters are critical regulatory elements involved in controlling transcription in cyanobacterial engineering. Both native and synthetic promoters have been employed to modulate gene expression within these organisms [97]. Native promoters, often sourced from cyanobacterial genes related to photosynthesis, carbon metabolism, or stress responses, are frequently used due to their compatibility with the host’s transcriptional machinery [98]. Notable examples include P*cp*c560, derived from the phycocyanin operon, and P*psbA2*, associated with photosystem II genes, both of which are widely used to drive gene expression in cyanobacterial systems [99]. In addition to native promoters, synthetic promoter libraries have been developed to enable more precise regulation of transcriptional activity. These engineered promoter sequences permit researchers to vary gene expression over a broad spectrum of strengths, thereby supporting the optimisation of multi-enzyme metabolic pathways. Synthetic promoters are especially useful in metabolic engineering contexts where careful adjustment of multiple gene expression levels is necessary for efficient pathway function [100,101].

Inducible gene expression systems have been established in cyanobacterial hosts, allowing precise control over gene activity in response to specific chemical or environmental cues. These systems facilitate temporal regulation of heterologous protein production [102,103]. Various inducible promoter systems have been adapted for cyanobacteria, including metal-responsive promoters such as P*petE*, which reacts to copper levels, and sugar-responsive systems like P*rhaBAD*, activated by rhamnose presence [104,105]. Such regulatory tools enable researchers to control the timing of gene expression, thereby minimising potential metabolic burdens on the host organism during enzyme expression.

In addition to transcriptional regulation, post-transcriptional elements such as ribosome binding sites [RBS] and translational control sequences are frequently utilised to refine gene expression [106]. By modulating RBS strength or altering mRNA stability, researchers can precisely adjust protein synthesis levels and enhance the efficiency of engineered metabolic pathways. Recent advances include the development of computationally designed RBS libraries and modular expression systems, which have significantly improved the ability to control protein expression in cyanobacterial hosts [100,106]. The combination of promoter engineering, inducible expression systems, and translational control elements, therefore, provides a versatile toolkit for the regulation of gene expression within cyanobacterial synthetic biology [96].

In addition to transcriptional and translational regulation, post-translational regulation is an important but often underused layer of gene-expression control in cyanobacterial engineering. Protein abundance and activity are shaped by folding efficiency, cofactor incorporation, disulfide-bond formation, proteolytic turnover and stress-responsive quality-control systems. In cyanobacteria, proteostasis is strongly influenced by ATP-dependent proteases such as the Clp system, which participates in protein quality control and degradation of damaged or misfolded proteins [107]. This is relevant for heterologous pathways because unstable enzymes may be rapidly degraded even when transcription and translation are efficient. Conversely, controlled degradation can be engineered deliberately using degron-based systems, providing a post-translational strategy to tune enzyme abundance, remove toxic pathway proteins or dynamically regulate metabolic flux [108]. For proteins requiring oxidative folding, disulfide-bond formation must also be considered, particularly when enzymes or recombinant products are targeted to the periplasm or extracellular space. The cyanobacterial SynDsbAB system has been shown to support extracytoplasmic disulfide-bond formation in oxygenic photosynthetic organisms, highlighting the importance of redox-compatible localisation for disulfide-containing proteins [109]. Therefore, cyanobacterial gene-expression networks should be viewed as multilayered systems integrating promoter strength, translation efficiency, protein folding, maturation, localisation and turnover.

Beyond small-molecule inducible promoters, three additional classes of dynamic regulatory elements have been developed for cyanobacteria that expand the precision and flexibility of gene expression control. Light-responsive regulation is particularly well suited to cyanobacteria given their photoautotrophic lifestyle. While native promoters such as P*psbA* and P*cpcB* respond to light, their use is complicated by direct coupling to photosynthetic activity and variable expression across light intensities. Optogenetic systems offer a more precise alternative: the two-component CcaS/CcaR system, originally characterised in *Synechocystis* sp. PCC 6803, senses green and red light wavelengths and has been successfully implemented in *Synechococcus* sp. PCC 7002 to achieve reversible, tuneable gene expression with a 6-fold dynamic range, enabling the light supply itself to serve as an orthogonal inducer signal without chemical addition [110]. Riboswitches represent a complementary post-transcriptional regulatory layer operating entirely at the RNA level through ligand-induced conformational changes in the 5′ untranslated region of mRNAs; theophylline-responsive riboswitches have been demonstrated to regulate gene expression across multiple phylogenetically diverse cyanobacterial species including *Synechococcus elongatus* PCC 7942, *Anabaena* sp. PCC 7120, and non-model strains, and offer several practical advantages—no additional protein components required, rapid response, and minimal metabolic burden [103]. Quorum-sensing circuits provide a third regulatory dimension, enabling density-dependent gene expression that can be used for autonomous production switching or cross-species communication in consortia; Kokarakis et al. demonstrated the first implementation of heterologous acyl-homoserine lactone quorum-sensing pathways (Lux, Las, and Tra) in *S. elongatus* PCC 7942, showing that these circuits detect AHL signals in a dose-dependent manner and can link *E. coli* population density to cyanobacterial gene expression outputs, a framework directly applicable to regulating sucrose secretion and other carbon transfer events in synthetic co-cultures [111]. Together these three regulatory modalities—light-responsive, riboswitch-based, and quorum-sensing—substantially extend the toolkit for precise, dynamic, and context-dependent gene regulation in cyanobacterial metabolic engineering.

### 3.4. CRISPR-Based Genome Editing in Cyanobacteria

In recent years, CRISPR-based genome editing technologies have emerged as formidable tools for precise genetic manipulation across various organisms, including cyanobacteria [112]. Originating from bacterial adaptive immune systems, these mechanisms facilitate targeted DNA modifications by employing programmable guide RNAs that direct CRISPR-associated nucleases to specific genomic sequences. The development of CRISPR technologies has significantly improved the efficiency and accuracy of genome engineering relative to traditional homologous recombination techniques [113,114].

Among the CRISPR systems employed for cyanobacterial engineering, CRISPR–Cas9 and CRISPR–Cas12a [Cpf1] have received considerable attention. These nucleases facilitate site-specific double-stranded DNA breaks at designated loci, which are subsequently repaired via homologous recombination utilising supplied donor DNA templates [115,116]. Cas9 and Cas12a differ in several mechanistic features, including their guide RNA structures, protospacer-adjacent motif [PAM] requirements, and cleavage patterns. Cas9 typically generates blunt-ended double-stranded DNA breaks, whereas Cas12a produces staggered DNA breaks with short overhangs. These differences can influence target-site availability, repair outcomes, and the design of editing strategies in cyanobacterial hosts. Early demonstrations of CRISPR-mediated genome editing in cyanobacteria include the work of Ungerer and Pakrasi, who successfully applied a CRISPR–Cpf1 system for targeted genomic modifications in *Synechococcus elongatus* UTEX 2973 [117]. Such approaches enable precise gene knockouts, insertions, and replacements, thereby enhancing the capacity for metabolic pathway engineering with increased accuracy and efficiency. Notably, Cas12a-based systems have emerged as particularly advantageous in cyanobacterial applications due to their capacity to process multiple guide RNAs from a single transcript, enabling multiplex genome editing and concurrent manipulation of several genetic targets [115,116].

Besides genome editing, CRISPR technology has also been adapted for transcriptional regulation via CRISPR interference [CRISPRi]. In this method, a catalytically inactive Cas protein [dCas] is directed to specific genomic sites where it obstructs transcription without causing DNA breaks [118]. Yao et al. demonstrated the use of CRISPRi in cyanobacteria for targeted gene repression, offering a strategic tool for modulating gene expression and redirecting metabolic fluxes within native networks. This approach provides a valuable means for studying gene function and fine-tuning of metabolic pathways without permanent genomic alterations [119,120].

Despite the benefits of CRISPR-based methods, several challenges persist in their application in cyanobacterial systems. Factors such as DNA repair pathways, transformation efficiency, and potential toxicity from nuclease expression can impact editing outcomes, requiring careful optimisation of experimental conditions. Nonetheless, ongoing advancements in CRISPR technology are broadening the scope of cyanobacterial genetic engineering and are poised to play a progressively significant role in developing engineered photosynthetic biotechnologies [116,121]. Recent advances have expanded cyanobacterial genome editing beyond standard nuclease-based CRISPR toward marker-free and single-nucleotide precision strategies. Marker-free multiplex genome editing via optimised CRISPR-Cas12a has been demonstrated in *Synechocystis* sp. PCC 6803 [122] and the endogenous Type I-D CRISPR-Cas system of *Synechococcus* sp. PCC 7002 has been repurposed for scarless gene deletions using native cyanobacterial machinery [123]. Complementing these approaches, cytosine base editing—which achieves precise C-to-T substitutions without double-strand breaks, donor DNA, or Cas nuclease toxicity—has been demonstrated in *Synechococcus elongatus* [124] and subsequently extended to *Synechocystis* and *Anabaena* with multiplex capability and integrated counter-selection for plasmid curing [125]. These tools directly address the segregation delays and marker accumulation constraints that have historically slowed complex genome engineering in polyploid cyanobacterial strains.

Prime editing, which enables all twelve base-to-base substitutions alongside small insertions and deletions without double-strand breaks or donor DNA, represents an emerging frontier for cyanobacterial genome engineering. Its full implementation in photosynthetic hosts is yet to be established but represents an important near-term direction for precise metabolic engineering applications.

**Figure 1:**
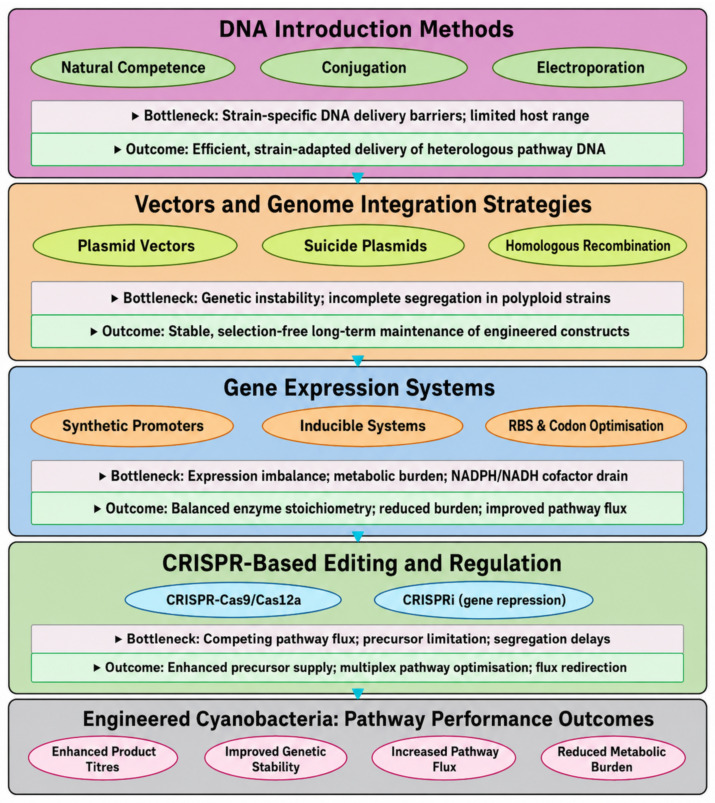
Overview of genetic tools available for different steps involved in cyanobacterial pathway engineering.

A summary of the major genetic engineering tools currently available for cyanobacterial genome manipulation and metabolic engineering is provided in Table 1, and a simplified overview of the main genetic tools used for cyanobacterial engineering is presented in Figure 1.

Taken together, the genetic tools described in this section differ substantially in their suitability for specific strains, their effects on pathway stability, and their capacity to support expression control and product yield optimisation, and these differences should directly inform tool selection in metabolic engineering applications. With respect to strain compatibility, natural transformation is the method of choice for naturally competent strains such as *Synechocystis* sp. PCC 6803 and *Synechococcus elongatus* PCC 7942, enabling direct chromosomal integration without additional delivery systems, while conjugation from *E. coli* remains the most reliable approach for filamentous strains such as *Anabaena* sp. PCC 7120 and other strains with limited natural competence. Electroporation offers a rapid physical delivery alternative but produces variable transformation efficiencies across strains and requires extensive protocol optimisation. Regarding pathway stability, chromosomal integration via homologous recombination provides the greatest long-term genetic reliability, as integrated constructs are inherited without antibiotic selection pressure; however, the polyploid nature of most cyanobacterial strains means that complete segregation of engineered loci can be slow, producing mixed populations that compromise product yield consistency. Plasmid-based systems allow more rapid pathway prototyping but are prone to plasmid loss during extended cultivation without selection, making them less suitable for stable long-term production [10]. CRISPR–Cas9 and Cas12a systems improve targeted editing efficiency and enable multiplex manipulation of competing pathways, particularly advantageous for simultaneously disrupting carbon flux sinks, but Cas nuclease toxicity and incomplete segregation in polyploid strains remain practical limitations. With respect to expression control and product yield, synthetic promoter libraries and inducible systems such as P*petE* provide substantially greater regulatory precision than native promoters, enabling calibration of individual enzyme levels to minimise metabolic bottlenecks and reduce the accumulation of toxic intermediates. CRISPRi represents a particularly valuable approach for improving product yield through targeted repression of competing native pathways, enabling carbon flux redirection without permanent genomic disruption, though repression stability over extended cultivation requires validation [3]. Collectively, these considerations underscore that tool selection in cyanobacterial metabolic engineering is inherently strain- and application-specific, and that the interplay between transformation method, integration strategy, and expression control has direct consequences for the consistency and magnitude of product yields. More broadly, the choice of integration strategy directly influences product yield consistency—chromosomally integrated pathways in fully segregated strains produce more reproducible and stable yields than plasmid-based systems, while the combination of synthetic promoter tuning and CRISPRi-mediated repression of competing routes has been shown to produce the greatest gains in heterologous product titres in cyanobacterial systems.

## 4. Engineering Heterologous Metabolic Pathways in Cyanobacteria

The introduction of heterologous metabolic pathways is a key strategy in the field of cyanobacterial metabolic engineering. By integrating genes from other organisms, cyanobacteria can be engineered to produce compounds absent from their native metabolic networks [141,142]. This methodology has facilitated the development of photosynthetically powered microbial systems capable of transforming CO_2_ and sunlight into diverse chemical products through customised biosynthetic pathways [142].

Engineering heterologous pathways in cyanobacteria typically involves the coordinated expression of multiple genes encoding enzymes that convert native metabolic intermediates into desired compounds. The successful implementation of such pathways demands careful consideration of various factors, including gene expression levels, enzyme activity, metabolic flux distribution, and the availability of cellular cofactors [79,100]. Given that cyanobacterial metabolism is closely linked to photosynthetic energy generation and carbon fixation, integrating new biosynthetic pathways necessitates balancing these processes with the host’s native metabolism to preserve cellular growth and physiological stability [1].

A common approach in metabolic pathway engineering involves rerouting metabolic flux from central carbon pathways toward designed biosynthetic routes. In cyanobacteria, intermediates originating from the Calvin–Benson–Bassham cycle and subsequent metabolic processes such as glyceraldehyde-3-phosphate, pyruvate, and acetyl-CoA serve as essential precursors for heterologous biosynthesis [1]. Nonetheless, competition with native metabolic processes frequently restricts the efficiency of heterologous pathway activity. Therefore, optimisation efforts often include modifications to native pathways, deletion or repression of competing reactions, and enhancement of precursor availability to improve overall pathway performance [143]. For instance, Liu et al. demonstrated in *Synechocystis* sp. PCC 6803 that achieving the highest reported cyanobacterial 1-butanol titre of 4.8 g/L required simultaneous attention to all three of these dimensions: coordinated expression of the CoA-dependent pathway genes was optimised through systematic tuning of promoters and RBS sequences; carbon flux was rewired toward the acetyl-CoA precursor through modifications to central carbon metabolism; and competing pathways were repressed to redirect metabolic flux toward the target product - illustrating that no single intervention was sufficient and that all three must be addressed [144].

In addition to pathway design, effective heterologous expression necessitates meticulous optimisation of gene sequences and regulatory elements. Variations in codon usage between the source organism and the cyanobacterial host can influence translation efficiency, rendering codon optimisation a critical step when introducing foreign genes. Furthermore, the employment of suitable promoters, ribosome binding sites [RBS], and transcriptional regulators is crucial for ensuring balanced expression of multi-enzyme pathways. Managing the relative expression levels of pathway enzymes is particularly significant, as disproportionate enzyme abundance carries distinct mechanistic consequences for both pathway performance and host fitness. When upstream enzymes are overexpressed relative to downstream counterparts without a sufficient metabolic sink, reactive intermediates accumulate to concentrations that directly impair host growth; in *Synechocystis* sp. PCC 6803, overexpression of the MEP pathway enzymes DXS and IDI without a downstream terpenoid synthase caused significant growth impairment, which was relieved only when a carbon sink was present [145]. Cofactor drain is a further consequence specific to cyanobacteria: photosynthetic electron transport generates abundant NADPH but limited NADH, yet many heterologous enzymes from heterotrophic organisms require NADH as their primary reductant, creating a cofactor mismatch that simultaneously limits pathway flux and risks disrupting the NADPH/NADP^+^ balance required for carbon fixation [146]. At the level of host fitness, high-level expression of multiple heterologous proteins competes with native protein synthesis for ribosomes, amino acids, and ATP, diverting resources from photosynthetic maintenance and generating selective pressure for pathway-silencing mutations during prolonged cultivation [100,147].

These strategies collectively underpin heterologous metabolic pathway engineering in cyanobacteria. Progress in synthetic biology, genome editing, and systems-level metabolic analysis continues to enhance the capacity to design, build, and optimise engineered pathways [10]. Notably, modular pathway assembly techniques are increasingly employed to enable rapid testing and refinement of enzyme combinations and regulatory elements within cyanobacterial hosts. Such approaches allow researchers to iteratively improve pathway performance and increase the efficiency of engineered biosynthetic systems, thereby broadening the prospects for cyanobacteria as platforms in photosynthetic biotechnology [100].

### 4.1. Multi-Gene Pathway Construction and Design

The construction of heterologous metabolic pathways in cyanobacteria generally involves the coordinated expression of multiple genes that encode enzymes responsible for sequential biochemical reactions [33]. Designing such pathways demands careful attention to gene organisation, transcriptional regulation, and the compatibility of the introduced enzymes with the host cellular environment. In many instances, pathway genes are assembled into synthetic operons or modular expression cassettes, which enable the simultaneous expression of multiple enzymes within the cyanobacterial host [148].

Operon-based pathway design is frequently utilised in cyanobacterial engineering, particularly for expressing enzymes that act sequentially within a biosynthetic pathway. In such systems, multiple genes are regulated under a single promoter and co-transcribed as a polycistronic mRNA [149]. This setup facilitates coordinated transcription of pathway enzymes and simplifies the construction of genetic constructs. However, variations in translation efficiency among genes within an operon can affect the relative levels of individual enzymes, potentially impacting pathway efficiency [149]. Consequently, optimisation strategies often include modifying RBS, altering gene order within the operon, or adding transcriptional terminators to balance expression and reduce unintended transcriptional interactions [150].

Advances in synthetic biology have also enabled the development of modular DNA assembly techniques that allow rapid construction of multi-gene pathways. Methods such as Golden Gate cloning, Gibson assembly, and other modular cloning systems enable researchers to assemble multiple genetic elements including promoters, coding sequences, regulatory regions, and terminators into complex expression constructs with high efficiency [26,151]. These approaches have significantly expedited the design-build-test cycle in cyanobacterial metabolic engineering by simplifying the construction and iterative optimisation of engineered pathways [15].

Another important consideration in pathway design is ensuring the functional compatibility of heterologous enzymes within the cellular environment of cyanobacteria. Enzymes from different organisms can vary in their optimal temperatures, pH, cofactor dependencies, and protein-folding [primary, secondary, tertiary, or quaternary] properties [152]. Therefore, implementing heterologous pathways often necessitates additional optimisation steps such as codon optimisation, enzyme engineering, or the co-expression of auxiliary proteins that facilitate proper folding or activity. Sometimes, it may also be necessary to fine-tune enzyme expression levels or modify gene copy numbers to prevent metabolic bottlenecks and promote efficient pathway function [26]. Recent advances in synthetic biology have further explored the spatial organisation of pathway enzymes through synthetic scaffolds or protein interaction domains, aiming to enhance metabolic flux by positioning enzymes in close proximity and thereby improving substrate channelling within engineered pathways [153].

Overall, the design and assembly of multi-gene metabolic pathways are fundamental aspects of cyanobacterial synthetic biology. Attention to gene organisation, regulatory elements, enzyme compatibility, and pathway balance is crucial for ensuring the effective functioning and stability of engineered biosynthetic systems within cyanobacterial hosts [141,142].

### 4.2. Optimising Gene Expression and Metabolic Flux

Following the introduction of heterologous metabolic pathways, optimising gene expression and metabolic flux is essential for the effective operation of engineered biosynthetic pathways. In cyanobacterial models, precise regulation of individual enzyme expression levels is critical to ensure the efficient conversion of metabolic intermediates at each step of the biosynthetic pathway. Imbalances in enzyme expression can result in the accumulation of intermediates, decreased pathway efficiency, or increased metabolic burden on the host organism [154,155]. A common strategy for improving pathway performance involves fine-tuning transcriptional and translational regulatory elements to accurately regulate enzyme expression levels. Adjustments to promoter strength, modifications to ribosome binding site sequences, and optimisation of gene copy number are techniques that affect protein expression within the cyanobacterial host cell. By systematically varying these regulatory components, researchers can identify gene expression configurations that enhance metabolic fluxes in cyanobacterial engineered pathways [156].

Another critical factor affecting the performance of heterologous pathways is the availability of metabolic precursors stemming from central carbon metabolism. The competition between native metabolic processes and introduced pathways can thereby restrict the supply of precursor molecules essential for pathway function. To mitigate this issue, metabolic engineering approaches often aim to redirect metabolic flux toward desired biosynthetic routes by modifying or repressing competing pathways [118].

Co-factor availability significantly influences pathway efficiency [157]. Numerous biosynthetic enzymes rely on reducing equivalents such as NADPH, as well as cofactors including ATP, flavins, and metal ions, to function properly. Since cyanobacterial metabolism is closely linked to photosynthetic energy production, variations in light intensity and photosynthetic activity can impact the intracellular levels of these cofactors [79,158]. Notably, the ratio of ATP to NADPH produced during PET can influence the efficacy of engineered pathways that are heavily dependent on reducing power. Therefore, optimising pathways may necessitate modifications in enzyme selection, regulation, or cultivation conditions to ensure sufficient cofactor availability [155,159].

In cyanobacteria, metabolic burden is therefore not only a consequence of expressing additional proteins, but also reflects competition for photosynthetically generated energy and reducing power. Linear photosynthetic electron transport transfers electrons from water through PSII, the plastoquinone pool, cytochrome b_6_f, PSI, ferredoxin and ferredoxin–NADP^+^ reductase, producing ATP through proton translocation and NADPH through reduction in NADP^+^. These ATP and NADPH pools are normally partitioned toward the Calvin–Benson–Bassham cycle, carbon-concentrating mechanisms, nitrogen assimilation, stress responses and cellular maintenance. The Calvin cycle itself requires a high ATP/NADPH input, with an approximate ATP/NADPH demand of 1.5 for CO_2_ fixation [160]. However, linear electron flow does not provide unlimited flexibility in this ratio, and cyanobacteria rely on cyclic and pseudo-cyclic electron transport routes to adjust ATP generation, dissipate excess reducing power and maintain redox poise [161]. When a heterologous pathway is introduced, it creates an additional ATP and/or NADPH sink. If this sink is poorly matched to photosynthetic electron output, the result can be reduced carbon fixation, over-reduction or oxidation of electron carriers, impaired growth, increased reactive oxygen species formation and lower product yield. This is particularly important for redox- and cofactor-intensive pathways such as alcohol, lactate, terpenoid, fatty acid or cytochrome P450-dependent biosynthesis, where pathway flux can become limited by electron supply rather than by gene expression alone. Thus, the core limitation is often the partitioning of photosynthetic ATP, NADPH and reduced ferredoxin between native metabolism and the engineered pathway, rather than metabolic burden in a general sense. Therefore, effective pathway design in cyanobacteria should consider not only precursor availability and enzyme expression, but also the stoichiometric demand for ATP, NADPH, NADH and reduced ferredoxin relative to the photosynthetic energy budget of the host.

Together, these strategies highlight the importance of carefully balancing target enzyme expression, required precursor availability, and cofactor supply in engineering heterologous metabolic pathways within cyanobacteria. A systematic approach to rational pathway design in cyanobacteria integrates multi-omics data—spanning transcriptomics, proteomics, metabolomics, and fluxomics—with genome-scale metabolic models (GEMs) to achieve a systems-level understanding of how heterologous pathways interact with native phototrophic metabolism GEMs of cyanobacteria, most extensively developed for *Synechocystis* sp. PCC 6803, represent complete reconstructions of the metabolic network that can be interrogated using flux balance analysis to predict intracellular flux distributions, identify competing metabolic sinks, and calculate theoretical yield limits under photoautotrophic conditions [78,162]. When constrained by transcriptomic or proteomic data, these models become condition-specific, enabling more accurate identification of bottlenecks that would not be apparent from any single data layer alone [163]. The integration of multi-omics data with GEMs and machine learning has further demonstrated that cross-omic features explain cyanobacterial metabolic adaptation to fluctuating light and nutrient conditions that transcriptomics alone cannot reveal, illustrating the power of this combined approach for informing rational engineering decisions [164].

### 4.3. Codon Optimisation and Heterologous Protein Expression

The efficient expression of heterologous enzymes is essential for the successful implementation of engineered metabolic pathways in cyanobacteria [3]. When genes derived from phylogenetically distant organisms are introduced into cyanobacterial hosts, differences in codon usage can markedly affect translation efficiency and thus protein yield [26]. Codon bias refers to the preferential use of specific synonymous codons within a genome, and heterologous genes containing codons that are rarely used by the host may exhibit lower translation rates or incomplete protein synthesis. As a result, codon optimisation is commonly employed to modify gene sequences to better align with the codon usage preferences of the cyanobacterial host.

Beyond codon optimisation, efficient heterologous protein expression in cyanobacteria requires optimisation of the full expression process, including translation, folding, cofactor maturation, intracellular localisation and functional positioning of the enzyme. Heterologous enzymes may fold inefficiently in cyanobacterial cells because of differences in redox environment, temperature optimum, cofactor availability, oligomerisation requirements or electron-transfer partners. Misfolded proteins may be degraded or accumulate as inactive aggregates, meaning that high transcript abundance does not necessarily translate into high pathway activity. Therefore, expression strategies should also include RBS tuning, translational leader optimisation, N- or C-terminal fusion tags, removal of destabilising domains and, where appropriate, co-expression of molecular chaperones or maturation factors. This is particularly important for complex enzymes such as metalloenzymes, oxygen-sensitive enzymes and cytochrome P450 systems, which require correct folding, cofactor incorporation and compatible electron-transfer partners. In *Synechocystis* sp. PCC 6803, systematic optimisation of heterologous expression for α-bisabolene production showed that codon optimisation, expression-cassette design and translational tuning can strongly influence protein accumulation and product titres [165].

Post-translational regulation should also be considered during heterologous protein optimisation. Chaperone co-expression can improve solubility and assembly of difficult proteins, particularly multi-subunit or aggregation-prone enzymes; for example, DnaK/DnaJ-family chaperones from *Synechocystis* sp. PCC 6803 have been shown to support soluble Rubisco assembly in a heterologous system [166]. For disulfide-containing enzymes or recombinant proteins, expression may require targeting to a compatible oxidative environment, such as the periplasm, together with appropriate use of the Sec/Tat translocation route and endogenous disulfide-bond machinery. At the same time, unstable heterologous proteins may be recognised by cellular quality-control systems and degraded, reducing active enzyme levels. Strategies such as removing degradation-prone regions, using stabilising fusion tags, controlling expression strength, co-expressing chaperones or designing regulated degrons can therefore help balance protein stability with pathway activity.

Intracellular localisation and membrane anchoring provide an additional level of control over heterologous pathway performance. Although cyanobacteria lack eukaryotic organelles, their cells are highly organised and contain thylakoid membranes, carboxysomes, the periplasm and distinct cytoplasmic microenvironments. Heterologous enzymes can therefore be directed to specific cellular locations using signal peptides, secretion tags, membrane-targeting sequences or protein-fusion strategies. Sec- and Tat-dependent translocation pathways are particularly relevant for targeting proteins to the periplasm or extracellular space; Sec generally transports unfolded proteins, whereas Tat can transport folded proteins, making pathway selection important for enzyme activity and stability [167]. For membrane-associated or electron-transfer-dependent enzymes, thylakoid anchoring can position heterologous proteins close to photosynthetic electron transport components, ferredoxin and NADPH-generating systems. This approach has recently been shown to improve the stability and activity of a heterologous cytochrome P450 in *Synechocystis* sp. PCC 6803 [168]. In addition, synthetic scaffolds and self-assembling protein structures can be used to co-localise sequential pathway enzymes, increase local substrate concentration and improve metabolic channelling, as demonstrated by nanofilament-based protein co-localisation systems in cyanobacteria [169]. Together, these strategies show that heterologous protein optimisation in cyanobacteria should consider codon usage, translation, folding, cofactor maturation, localisation and membrane anchoring as connected design parameters.

These factors highlight the importance of carefully designing heterologous gene constructs to achieve optimal protein expression and enzymatic activity in cyanobacterial hosts. Ongoing advancements in synthetic biology and gene engineering techniques continue to enhance the capacity to optimise heterologous enzyme expression, thereby improving the functionality of engineered metabolic pathways in cyanobacteria [79].

### 4.4. Cofactor Balancing and Precursor Supply

The efficient functioning of heterologous metabolic pathways in cyanobacteria relies not only on enzyme expression but also on the availability of metabolic precursors and intracellular cofactors essential for biosynthetic processes [26]. Since cyanobacterial metabolism is closely linked to photosynthetic carbon fixation, many engineered pathways depend on intermediates originating from the Calvin–Benson–Bassham cycle and related central metabolic routes. Key metabolites such as glyceraldehyde-3-phosphate, pyruvate, and acetyl-CoA often serve as precursors for heterologous biosynthesis. Therefore, the distribution and abundance of these intermediates within the metabolic network can significantly impact the performance of engineered pathways [1].

The competition between native metabolic processes and introduced biosynthetic pathways frequently restricts the availability of precursor metabolites. In cyanobacteria, carbon fixed through the Calvin cycle is typically allocated toward biomass production, including carbohydrate synthesis, amino acid formation, and cellular maintenance [170]. Approaches like overexpressing key enzymes involved in central carbon metabolism, knocking out competing pathways, or modifying carbon partitioning mechanisms have been investigated to enhance precursor supply for heterologous biosynthesis [144].

Apart from precursor availability, cofactor balance is a significant factor affecting pathway efficiency [171]. Many biosynthetic enzymes depend on reducing equivalents such as NADPH or cofactors including ATP, flavins, or metal ions to carry out their reactions. In cyanobacteria, the intracellular levels of ATP and NADPH are closely tied to PET. Consequently, variations in light intensity, carbon sources, or environmental conditions can alter the cell’s redox state. This dynamic means that engineered pathways demanding substantial reducing power may compete with native metabolic processes for these cofactors, thereby potentially constraining overall pathway performance [172].

Addressing these challenges necessitates strategies that enhance cofactor availability or rebalance cellular redox metabolism. Approaches such as modifying electron transport pathways, introducing enzymes with alternative cofactor specificities, or adjusting cultivation conditions to improve photosynthetic efficiency have been investigated as methods to increase cofactor supply in cyanobacteria. Furthermore, advancements in systems biology including metabolic flux analysis, transcriptomics, and genome-scale metabolic modelling are increasingly employed to identify metabolic bottlenecks and inform rational engineering strategies aimed at optimising precursor distribution and cofactor utilisation within cyanobacterial cells [173].

These considerations highlight the importance of integrating pathway design with comprehensive metabolic network engineering in the development of cyanobacterial production systems. Ensuring sufficient precursor availability and cofactor balance is crucial for the effective functioning of heterologous metabolic pathways and for enhancing the overall performance of engineered cyanobacterial strains [8].

### 4.5. Examples of Successful Heterologous Metabolic Pathways in Cyanobacteria

Numerous studies have established that cyanobacteria can be genetically engineered to support functional heterologous metabolic pathways, enabling the production of fuels, platform chemicals, and high-value compounds. These findings are significant because they demonstrate that cyanobacteria are not only candidate photosynthetic hosts but can also convert carbon fixed through photosynthesis into non-native products via the introduction of specific biosynthetic pathways.

Xie et al. [174] demonstrated the successful engineering of *Synechocystis* sp. PCC 6803 for the photosynthetic production of isobutanol and 3-methyl-1-butanol via a synthetic 2-keto acid pathway. This research indicated that pathway efficiency could be enhanced through metabolic engineering strategies, such as increasing the gene copy number within the pathway and overexpressing specific genes involved in central carbon metabolism. Long-term cultivation of the engineered strains yielded cumulative titres of 1247 mg/L isobutanol and 389 mg/L 3-methyl-1-butanol, underscoring the potential of cyanobacteria for direct production of advanced alcohols from photosynthetically fixed carbon. While these cyanobacterial titres represent meaningful progress, they remain substantially lower than those achieved in heterotrophic hosts. Atsumi et al. [175] produced isobutanol at 22 g/L in *E. coli* from glucose, reaching 86% theoretical yield while Liang et al. [176] achieved 13.67 g/L at 0.456 g/L/h in a 30 h batch fermentation, and *S. cerevisiae* has yielded up to 14.8 g/L from xylose [177]. The cyanobacterial cumulative titre of 1247 mg/L over 58 days equates to approximately 21.5 mg L^−1^ d^−1^ average daily productivity, compared to approximately 10,900 mg L^−1^ d^−1^ for *E. coli*—a several-hundred-fold difference reflecting light-dependent growth constraints rather than poor pathway design.

For phototrophic systems, specific productivity and areal productivity are more informative benchmarks than volumetric titre alone. Based on the maximum OD750 of 5.95 reported in the same study and a standard DCW conversion factor of ~0.25 g L^−1^ per OD750 unit for *Synechocystis* sp. PCC 6803, the estimated specific isobutanol productivity is approximately 21 mg g DCW^−1^ d^−1^. Areal productivity cannot be calculated from flask-scale data as illuminated surface areas are not reported. Industrially relevant isobutanol production targets titres exceeding 20 g/L and productivities above 1 g L^−1^ h^−1^; current cyanobacterial values remain one to two orders of magnitude below these thresholds, though the use of CO_2_ and light rather than organic carbon inputs changes the sustainability basis of the comparison considerably [15].

Another notable example is the genetic engineering of *Synechocystis* sp. PCC 6803 for the biosynthesis of 1-octanol. Research by Yunus et al. [178] demonstrated that cyanobacteria can be modified to produce this medium-chain alcohol, which holds promise as both a chemical precursor and a biofuel component. By optimising metabolic pathways and refining cultivation conditions, the engineered strains reached a 1-octanol titre of 526 ± 5 mg/L after 12 days, with carbon partitioning efficiency reaching approximately 35%. This case highlights both the potential and the challenges associated with introducing heterologous alcohol biosynthesis pathways into photosynthetic organisms For context, engineered *E. coli* strains have achieved 1-octanol titres of up to 1.3 g/L with greater than 90% C8 selectivity from glycerol in batch fermentations of 24–48 h [179], representing an approximately 2.5-fold higher titre than the cyanobacterial result but achieved from an organic carbon feedstock in a fraction of the cultivation time. On a daily productivity basis the gap widens considerably, and current cyanobacterial 1-octanol production remains well below industrially relevant thresholds, highlighting that medium-chain fatty alcohol biosynthesis in phototrophic hosts is constrained by the same light-dependent growth limitations that restrict other cyanobacterial product classes.

A more recent example involves the production of taxol [a chemotherapy drug] precursors in *Synechocystis* sp. PCC 6803. Zhong et al. [180] engineered modular heterologous pathways that incorporated methylerythritol phosphate pathway enzymes alongside taxol biosynthetic enzymes, enabling the production of oxygenated taxanes, including taxadiene-5α-ol, directly from carbon dioxide. The most effective strain yielded 17.43 mg/L of oxygenated taxanes and 4.32 mg/L of taxadiene-5α-ol, marking the first instance of photosynthetic taxadiene-5α-ol synthesis from CO_2_ in cyanobacteria. This research is particularly noteworthy because it advances cyanobacterial heterologous pathway engineering beyond simple alcohols and platform chemicals, toward the biosynthesis of more complex, high value terpenoids. For comparison, engineered *Saccharomyces cerevisiae* has achieved taxadiene titres of up to 184 mg/L in fed-batch bioreactor cultivation [181], approximately 10-fold higher on a volumetric basis than the cyanobacterial oxygenated taxane titres reported by Zhong et al. However, the cyanobacterial result is notable in producing hydroxylated oxygenated taxanes including taxadiene-5α-ol directly from CO_2_—a biosynthetically more advanced product than the unhydroxylated taxadiene typically achieved in heterotrophic hosts—demonstrating that cyanobacterial systems can access complex multi-step terpenoid pathways from inorganic carbon inputs, even if current titres remain below industrially relevant level.

Beyond alcohols, terpenoids and recombinant proteins, cyanobacteria have also been explored as heterologous hosts for structurally complex natural products, including alkaloids and flavonoid-related compounds. In alkaloid biosynthesis, *Anabaena* sp. PCC 7120 has been developed as a heterologous expression host for cyanobacterial indolactam pathways. Videau et al. expressed the lyngbyatoxin A biosynthetic genes in *Anabaena* sp. PCC 7120 and demonstrated production of lyngbyatoxin A at levels comparable to those reported for the native producer, *Moorea producens*; importantly, the study also showed that titres could be improved through genetic and promoter-level optimisation [182]. This system was subsequently expanded as a combinatorial platform for related indolactam natural products, where codon-optimised genes enabled the production of lyngbyatoxin A, pendolmycin and teleocidin B-4 in *Anabaena* sp. PCC 7120, demonstrating that cyanobacteria can support more complex natural product biosynthesis beyond simple linear pathways [183]. A further example is the engineered production of hapalindole-type alkaloids in the fast-growing cyanobacterium *Synechococcus elongatus* UTEX 2973. Knoot et al. reconstructed core genes from the approximately 42 kb *fam* hapalindole biosynthetic gene cluster of *Fischerella ambigua* UTEX 1903 into synthetic operons, enabling the production of indole-isonitrile intermediates and the hapalindole alkaloids hapalindole H and 12-epi-hapalindole U at titres of approximately 0.75–3 mg/L [184]. These examples show that cyanobacterial hosts can accommodate multi-enzyme alkaloid biosynthetic pathways and can be used not only for production, but also for pathway reconstruction and combinatorial biosynthesis.

Cyanobacteria have also been engineered for plant phenylpropanoid and flavonoid-related metabolism. Xue et al. engineered *Synechocystis* sp. PCC 6803 by introducing the tyrosine ammonia lyase gene *sam8* from *Saccharothrix espanaensis* and deleting the native *slr1573* laccase-like gene, resulting in secretion of approximately 82.6 mg/L p-coumaric acid into the culture medium [185]. Although *p*-coumaric acid is not itself a flavonoid, it is a central phenylpropanoid intermediate that can be converted through *p*-coumaroyl-CoA and chalcone intermediates into flavonoid structures. More directly, Ni et al. developed a photoautotrophic cyanobacterial platform in *Synechococcus elongatus* PCC 7942 for the production of plant natural products directly from CO_2_, including *p*-coumaric acid, caffeic acid, ferulic acid, resveratrol, naringenin and bisdemethoxycurcumin, with reported titres ranging from 4.1 to 128.2 mg/L [186]. The production of naringenin is particularly relevant because it represents a core flavanone scaffold and a precursor for multiple downstream flavonoid classes. These studies broaden the application scope of cyanobacterial metabolic engineering by showing that photosynthetically fixed carbon can be redirected not only into fuels, terpenoids and recombinant proteins, but also into aromatic amino acid-derived and malonyl-CoA-dependent natural product pathways. At the same time, they highlight important remaining bottlenecks for complex natural product biosynthesis in cyanobacteria, including precursor competition, malonyl-CoA availability, pathway enzyme compatibility and the need to balance heterologous pathway demand with photosynthetic ATP and NADPH supply [187].

Beyond small-molecule biosynthesis, cyanobacteria have also been explored as photosynthetic platforms for heterologous recombinant protein production, including therapeutic proteins, vaccine antigens and antibody fragments. For example, Betterle et al. engineered *Synechocystis* sp. PCC 6803 to express human interferon α-2, a therapeutic cytokine with antiviral and anti-proliferative activity, as a fusion protein; the recombinant protein accumulated in soluble form and showed preliminary antiviral activity [188]. Cyanobacteria have also been investigated for vaccine-related protein delivery, with *Anabaena*-expressed VP28 used for oral administration against white spot syndrome virus in shrimp, and transgenic *Synechococcus elongatus* PCC 7942 further developed to express a VP28–mOrange fusion protein for traceable oral delivery studies [189,190]. More recently, Kasai et al. demonstrated the production of functional anticancer antibody fragments in *Synechocystis* sp. PCC 6803, including scFv, VHH and bispecific tandem VHH formats, with the bispecific VHH retaining target-binding and cytotoxic activity [191].

Beyond single-organism pathway engineering, an emerging approach positions cyanobacteria not as complete biosynthetic factories but as photosynthetic carbon donors within synthetic microbial consortia, where a heterotrophic partner performs the downstream biosynthetic conversion. This division of metabolic labour exploits the capacity of cyanobacteria to fix CO_2_ and export photosynthate, while delegating complex biosynthetic tasks to heterotrophic organisms with more mature genetic toolkits. The conceptual foundation for this approach was established by Ducat et al., who demonstrated that *Synechococcus elongatus* PCC 7942 engineered to express the sucrose/proton symporter CscB could export up to 80% of photosynthetically fixed carbon as sucrose, sufficient to support growth of co-cultivated *E. coli* and *Saccharomyces cerevisiae* without added organic carbon [192]. Weiss et al. subsequently developed a light-driven consortium pairing sucrose-exporting *S. elongatus* CscB with *Halomonas boliviensis*, achieving PHB productivities of up to 28.3 mg L^−1^ d^−1^ with production stability maintained for over five months without antibiotic selection [193]. Löwe et al. demonstrated a similar two-module design pairing *S. elongatus* CscB with *Pseudomonas putida* for photoautotrophic PHA production from CO_2_ [194]. This framework has since been extended to ethylene, isoprene, 3-hydroxypropionic acid, and 2,3-butanediol, typically using sucrose-secreting *Synechococcus* strains paired with engineered *E. coli* [195]. Key remaining challenges include managing co-culture population stability, controlling species ratios under light-limited conditions, and scaling beyond laboratory photobioreactors.

**Figure 2:**
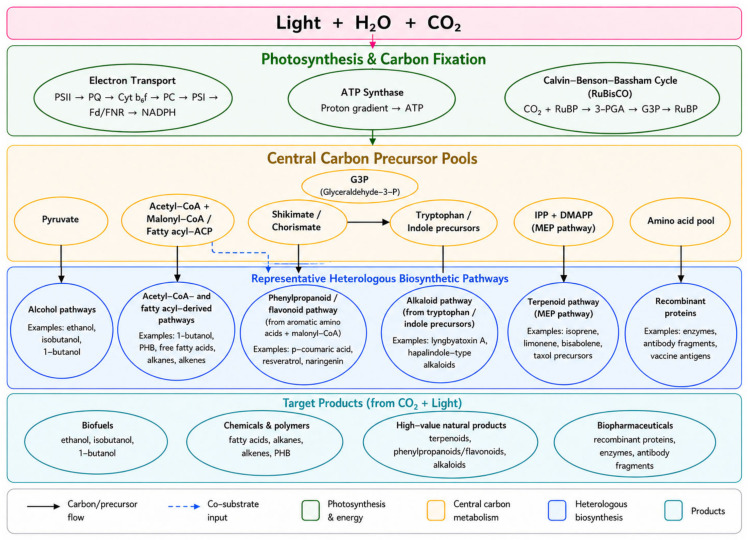
Metabolic network overview of heterologous biosynthetic pathways in engineered cyanobacteria. Abbreviations: PSII, photosystem II; PQ, plastoquinone; Cyt b_6_f, cytochrome b_6_f complex; PC, plastocyanin; PSI, photosystem I; Fd, ferredoxin; FNR, ferredoxin–NADP^+^ reductase; NADPH, nicotinamide adenine dinucleotide phosphate; ATP, adenosine triphosphate; RuBP, ribulose-1,5-bisphosphate; 3-PGA, 3-phosphoglycerate; G3P, glyceraldehyde-3-phosphate; IPP, isopentenyl diphosphate; DMAPP, dimethylallyl diphosphate; MEP, 2-C-methyl-D-erythritol 4-phosphate; PHB, poly(3-hydroxybutyrate).

These studies broaden the relevance of cyanobacterial engineering beyond fuel and chemical production, highlighting their emerging potential as sustainable hosts for recombinant biopharmaceutical production. These examples collectively highlight the flexibility of cyanobacteria as hosts for heterologous metabolic pathways. They also emphasise that successful pathway integration involves more than the mere introduction of foreign genes. The development of effective cyanobacterial production strains relies on meticulous pathway design, balanced enzyme expression, adequate precursor and cofactor availability, minimised metabolic burden, and sustained genetic and functional stability. Figure 2 presents a metabolic network map illustrating the relationships between photosynthetic carbon fixation, central precursor pools, heterologous biosynthetic pathways and the production of value-added compounds in engineered cyanobacteria.

## 5. Challenges and Limitations in Cyanobacterial Metabolic Engineering

Despite notable progress in genetic engineering and synthetic biology, various biological and technical obstacles continue to hinder the large-scale use of cyanobacteria as microbial production platforms. While cyanobacteria offer distinct advantages, such as photoautotrophic growth and the ability to fix CO_2_, they often exhibit lower productivity than traditional heterotrophic hosts like *Escherichia coli* or yeast. As a result, improving the efficiency, genetic stability, and metabolic capacity of engineered cyanobacterial strains remains a key focus in metabolic engineering research [196].

One of the most important limitations of cyanobacterial systems is their relatively slow growth rate compared to many heterotrophic microorganisms. Since cyanobacteria depend on photosynthetic energy production, biomass accumulation and product synthesis are inherently influenced by light availability and photosynthetic efficiency. As a result, their growth rates are often significantly lower than those of rapidly growing heterotrophic bacteria cultivated on organic carbon substrates. This slower growth can extend cultivation periods and diminish volumetric productivity, presenting challenges for industrial application [197]. Additionally, the introduction of complex heterologous metabolic pathways can elevate cellular metabolic demands, potentially exacerbating growth restrictions and further reducing overall productivity [198].

A central molecular basis of this metabolic burden is the competition for photosynthetically generated ATP and reducing power. In cyanobacteria, linear photosynthetic electron transport produces ATP and NADPH that are normally allocated to CO_2_ fixation through the Calvin–Benson–Bassham cycle, carbon-concentrating mechanisms, nitrogen assimilation, maintenance metabolism and stress responses. When heterologous pathways are introduced, they create additional ATP, NADPH, NADH or reduced ferredoxin demands that compete with these native processes. If pathway demand exceeds or poorly matches the photosynthetic energy and redox output of the host, carbon fixation can decline, electron carriers can become over-reduced or oxidised, reactive oxygen species may increase, and product formation becomes limited by cofactor availability rather than by enzyme expression alone. This mechanism is especially relevant for redox- and cofactor-intensive pathways such as alcohol, lactate, fatty acid, terpenoid and cytochrome P450-dependent biosynthesis. Therefore, targeted strategies such as cofactor-compatible enzyme selection, cofactor-specificity engineering, transhydrogenase introduction, re-routing of cyclic or pseudo-cyclic electron flow, and dynamic pathway regulation in response to light or redox status are needed to reduce energy competition and improve pathway stability [199].

Genetic instability during long-term cultivation is a major consequence of this metabolic burden. In engineered cyanobacteria, heterologous pathways can reduce host fitness by diverting carbon, ATP, NADPH, amino acids and transcriptional/translational capacity away from growth. Under prolonged batch, semi-continuous or outdoor cultivation, cells that acquire mutations reducing pathway expression may therefore grow faster than high-producing cells and gradually dominate the culture. At the molecular level, instability can arise through point mutations or indels in promoters, ribosome-binding sites, coding sequences or regulatory genes; deletion of pathway fragments through homologous recombination between repeated sequences; plasmid loss or copy-number reduction; transposon insertion; incomplete segregation of engineered alleles in polyploid genomes; or mutations that reduce expression of toxic or burdensome enzymes. These “escape” genotypes may retain normal growth but lose product formation, causing titres to decline over time even when the original engineered strain showed strong short-term production [200]. Long-term studies have also shown that production burden can favour the emergence of non-producing or low-producing cells during extended cultivation, emphasising the need to evaluate stability over many generations rather than relying only on early-stage titres [201]. Therefore, stable cyanobacterial strain design should minimise repeated DNA sequences, avoid unnecessary plasmid burden, use fully segregated chromosomal integration where possible, balance pathway expression to reduce toxicity, and include long-term monitoring of construct integrity, product titre and population heterogeneity.

An additional significant obstacle stems from the polyploid nature of many cyanobacterial genomes, which affects not only genome editing efficiency but also mutation segregation, genetic stability and engineered metabolic flux. Polyploidy is more than a technical obstacle in cyanobacterial genome editing; it directly influences genetic stability, mutation segregation and engineered metabolic flux. Many cyanobacteria contain multiple genome copies per cell, and ploidy level varies between species, strains and growth conditions. For example, *Synechococcus elongatus* PCC 7942 and marine *Synechococcus* WH7803 have been reported to contain approximately 3–4 genome copies per cell, whereas *Synechocystis* sp. PCC 6803 can be highly polyploid and shows strong growth-phase-dependent variation in genome copy number [202]. In *Synechocystis* sp. PCC 6803, ploidy is also influenced by external conditions such as light intensity and phosphate availability, indicating that genome copy number is a dynamic physiological property rather than a fixed strain characteristic [203]. Consequently, after homologous recombination, an engineered allele may initially be present in only some chromosome copies, while wild-type copies remain in the same cell. This creates partially segregated or merodiploid transformants and often requires repeated rounds of selection before a fully segregated engineered strain is obtained.

Incomplete segregation has important consequences for both genetic stability and metabolic performance. If the introduced pathway imposes a metabolic burden, cells retaining fewer engineered genome copies, or cells in which the construct is lost, silenced or mutated, may gain a growth advantage over high-producing cells. During prolonged cultivation, this can lead to enrichment of low-producing subpopulations and declining product titres. Polyploidy also affects gene dosage: different chromosomal or native plasmid integration sites can produce different expression levels because replicon copy number and local genomic context influence the number and activity of inserted gene copies [136]. As a result, partially segregated pathways may display unstable enzyme stoichiometry, variable precursor consumption and uneven demand for ATP, NADPH and carbon intermediates. Similarly, incomplete knockout of competing native pathways may leave residual wild-type activity, limiting the expected redirection of metabolic flux toward the target product.

Targeted engineering strategies are therefore needed to manage polyploidy rather than simply treating it as a background limitation. First, complete segregation should be confirmed using quantitative methods such as qPCR, digital PCR or genome sequencing, rather than relying only on endpoint colony PCR. Second, integration sites should be selected according to the required expression level and pathway burden; high-copy plasmid or native replicon sites may be useful for strong expression, whereas lower-copy chromosomal loci and weaker promoters may be preferable for toxic or cofactor-intensive pathways. Third, CRISPR-based systems can accelerate segregation by selectively cleaving unedited wild-type alleles and enriching fully edited genome copies. CRISPR-Cpf1/Cas12a systems and inducible CRISPR-Cas9 approaches have both been developed for cyanobacterial genome engineering and can help improve editing efficiency in polyploid hosts [115]. In addition, markerless and plasmid-free engineering strategies can improve long-term strain stability by avoiding repeated antibiotic-marker accumulation and reducing recombination between repeated selection cassettes When complete deletion of a competing pathway is destabilising, CRISPR interference can also provide a reversible strategy for reducing competing flux while maintaining essential growth functions.

Alongside genetic obstacles, efforts in metabolic engineering within cyanobacteria are often limited by constraints in metabolic flux and the availability of precursors. Numerous heterologous pathways compete with native cellular processes for carbon intermediates, ATP, and reducing agents such as NADPH [204]. Given that cyanobacterial metabolism is closely linked to photosynthetic energy production and cellular growth, redirecting carbon toward engineered biosynthetic pathways can place a metabolic strain on the host organism. This competition may result in diminished growth rates, reduced pathway efficiency, or instability of the engineered genetic constructs. Therefore, establishing a suitable balance between native metabolic functions and heterologous pathway activity remains a significant challenge in the engineering of cyanobacterial strains [204].

Finally, the long-term genetic stability of engineered pathways presents a significant challenge in the field of cyanobacterial metabolic engineering. The expression of heterologous enzymes can impose a metabolic burden on host cells, exerting selective pressure that favours mutations which diminish or eliminate pathway expression over prolonged cultivation periods. Such genetic instability may result in the gradual loss of engineered traits and diminished production yields over time [201]. To address this issue, strategies aimed at enhancing genetic stability—such as chromosomal integration of pathway genes and the implementation of balanced regulatory systems—are of critical importance in developing robust cyanobacterial production strains [50].

Cyanobacteria require inorganic nutrients for growth, primarily nitrogen, phosphorus, trace metals, and carbon dioxide, typically supplied as defined mineral media such as BG-11 in laboratory settings. While the individual components of these media are inexpensive, their costs become significant at the volumes required for large-scale production, and carbon dioxide supply—whether from compressed gas, bicarbonate, or flue gas—represents an additional upstream cost that life cycle analyses consistently identify as a major operating expense [205]. A techno-economic and life cycle analysis of cyanobacterial PHB production by Rueda et al. found a minimum selling price of 135 € kg^−1^ when using standard BG-11 medium, far exceeding the market price of approximately 4 € kg^−1^, and demonstrated that a volumetric productivity of at least 810 mg L^−1^ d^−1^ would be required to reach commercial viability—a threshold well beyond current reported values [205]. It should be noted that available techno-economic data are largely derived from biomass-retained compounds such as PHB, and the economics of volatile or secreted products may differ more favourably where in situ removal strategies avoid costly extraction steps. Substituting nutrient-rich wastewaters for defined synthetic media has also been explored as a strategy to reduce costs and environmental footprint, with some studies reporting reductions in cultivation costs of approximately 50%, though this approach introduces challenges regarding regulatory compliance and contamination risk for genetically engineered strains [206]. Artificial illumination represents a further major operating cost in enclosed photobioreactor systems, where electricity for lighting consistently constitutes one of the dominant energy expenditures; outdoor open raceway ponds substantially reduce this cost by utilising natural sunlight but introduce productivity variability and contamination risk. No system has yet achieved the productivity and economic competitiveness required for bulk chemical production. Biomass harvesting presents a third cost barrier, as cyanobacterial cells are small (1–5 µm) and of similar density to the surrounding medium; harvesting has been estimated to account for 20–30% of total operational costs, with centrifugation requiring 1–8 kWh m^−3^ and flocculation offering a cheaper though less consistent alternative at 0.1–6.7 kWh m^−3^ [207]. For volatile or secreted products such as isobutanol, in situ removal by gas stripping can bypass harvesting entirely, representing a meaningful economic advantage for these product classes. Overall, these challenges collectively demonstrate that successful metabolic engineering of cyanobacteria depends not only on developing advanced genetic tools but also on a thorough understanding of cyanobacterial physiology, metabolism, and cultivation needs. Overcoming these limitations is crucial for enhancing the efficiency and scalability of cyanobacterial production systems and unlocking the full potential of photosynthetic microorganisms in sustainable biotechnology [50]. A synthesis of the major cyanobacterial chassis strains discussed in this review, encompassing their available genetic tools, demonstrated product classes, key advantages, principal bottlenecks, and current technology readiness levels, is presented in Table 2.

## 6. Future Perspectives

The analysis presented in this review identifies five interconnected themes that represent both the primary unresolved challenges in cyanobacterial heterologous pathway engineering and the central conceptual contributions of this work: (i) balancing ATP and NADPH supply between photosynthetic energy generation and heterologous pathway demand; (ii) engineering cellular redox control to accommodate fluctuating light-driven cofactor availability; (iii) rationally directing heterologous enzyme localisation within the spatially organised cyanobacterial intracellular environment; (iv) employing synthetic scaffolds and metabolic channelling strategies to improve multi-enzyme pathway efficiency; and (v) ensuring long-term genetic stability of engineered constructs under the selective pressures imposed by extended photoautotrophic cultivation. Each of these themes is addressed in the following discussion, and collectively they define the engineering design principles that must be resolved if cyanobacterial heterologous pathway systems are to advance beyond laboratory demonstration toward scalable and reliable bioproduction.

Future developments will likely require moving beyond merely inserting heterologous genes towards more comprehensive pathway-engineering approaches that integrate enzyme expression, metabolic flux, host physiology, and long-term genetic stability as interconnected components [155,211].

An important future research direction involves the rational design of heterologous pathways through systems biology and computational modelling. Genome-scale metabolic models, flux analysis, and multi-omics datasets can be employed to predict interactions between introduced pathways and native carbon, energy, and redox metabolism [164]. These methods are particularly useful for identifying precursor limitations, competing metabolic sinks, and potential bottlenecks that may hinder product synthesis. In heterologous pathway engineering, such models can inform decisions regarding enzyme selection, modulation of native reactions, and redirection of carbon flux toward the target product, all while minimising adverse effects on cellular growth. This approach is especially critical for pathways that demand high levels of pyruvate, acetyl-CoA, glyceraldehyde-3-phosphate, ATP, or NADPH [212]. Recent advances have begun to realise this paradigm in practice: transcriptomic data has been integrated with the *Synechocystis* sp. PCC 6803 GEM to evaluate and improve constraint-based flux prediction methods under photoautotrophic conditions [163], while a dedicated GEM has been reconstructed for the fast-growing strain *Synechococcus* sp. PCC 11901, enabling dynamic flux balance analysis to identify production engineering targets in this high-productivity chassis [78]. Looking forward, the development of condition-specific cyanobacterial models—constrained by multi-omics data collected under actual production conditions rather than standard growth conditions—is expected to substantially reduce the number of experimental engineering cycles required to optimise heterologous pathway performance. Ultimately, the convergence of GEM-guided target identification, CRISPR-based genetic implementation, and multi-omics validation represents a complete design-build-test-learn cycle for cyanobacterial pathway engineering—a framework that is expected to progressively close the productivity gap between photosynthetic and heterotrophic production platforms as more comprehensive cyanobacterial datasets become available [173].

Another area of research involves optimising heterologous gene expression at the pathway level. Future investigations are expected to emphasise balancing the expression of individual pathway enzymes rather than simply maximising the expression of all components. Overexpression or poorly coordinated expression can impose metabolic burdens, lead to the accumulation of toxic intermediates, deplete cofactors, and reduce host viability [213]. Consequently, employing tuneable promoters, ribosome-binding site libraries, terminators, copy-number control, and carefully chosen genomic integration sites will be vital to achieving appropriate enzyme stoichiometry. The use of modular DNA assembly systems and standardised genetic parts will also facilitate more efficient design–build-test-learn cycles, allowing researchers to systematically evaluate diverse pathway architectures and identify configurations that enhance product yield and strain stability [151].

Dynamic regulation of heterologous pathways is a crucial future approach. Currently, many engineered pathways are expressed constitutively, which can divert cellular resources from growth and impose ongoing metabolic stress on the host organism. Implementing dynamic regulatory systems could enable modulation of pathway expression based on cellular conditions, light availability, carbon status, redox balance, or product accumulation [103]. For instance, growth-associated gene expression could be minimised during early biomass accumulation and activated during subsequent production phases. Such strategies may facilitate the decoupling of growth and production processes, reduce the metabolic burden of heterologous enzyme expression, and enhance overall productivity. This is especially pertinent in cyanobacteria, where photosynthetic energy generation and redox states are influenced by environmental fluctuations [214].

Future pathway engineering must also address cofactor and energy supply challenges more directly. Many heterologous biosynthetic pathways demand significant quantities of ATP, NADPH, NADH, flavin cofactors, or metal ions. Since cyanobacterial ATP and NADPH production is tightly linked to PET, pathway efficiency can be heavily affected by factors such as light intensity, carbon availability, and cellular redox balance [143]. Future strategies might involve engineering enzymes with alternative cofactor preferences, introducing transhydrogenase systems, redirecting photosynthetic electron flow, or modifying native cofactor-regeneration pathways. These approaches could enhance the compatibility between heterologous pathway requirements and cyanobacterial photosynthetic metabolism [215].

The intracellular organisation of heterologous pathways is also poised to become an increasingly important focus of research. In cyanobacteria, the spatial arrangement of photosynthetic membranes, carboxysomes, and central metabolic reactions creates an organised intracellular environment [169]. Future pathway design may benefit from targeting heterologous enzymes to specific subcellular locations, co-localising sequential enzymes, or employing synthetic scaffolds to improve metabolic channelling [169]. Such strategies could increase local substrate availability, minimise intermediate loss, improve enzyme stability, and boost pathway efficiency. This is especially relevant for complex multi-enzyme pathways, where performance depends not only on enzyme abundance but also on spatial proximity and access to substrates or cofactors [216].

Ensuring long-term genetic and functional stability will remain a key challenge in heterologous pathway development. Pathways that impose a high metabolic burden can create selective pressures for mutations that diminish or eliminate product formation. Consequently, future efforts should prioritise pathway designs that are both productive and stable over extended cultivation periods, particularly because non-producing cells may emerge and become enriched during scale-up or prolonged cultivation [217]. Approaches such as chromosomal integration, balanced expression systems, removal of unnecessary plasmid load, essential-gene coupling, and stability strategies without selection may contribute to more robust production strains. Additionally, evaluating pathway stability over prolonged cultivation, rather than relying solely on short-term product yields, will be important [217].

Adaptive laboratory evolution (ALE) should be applied as a targeted optimisation strategy rather than as a general growth-improvement tool. A systematic ALE scheme for cyanobacterial pathway engineering should first define the desired phenotype, such as improved product tolerance, tolerance to high light or fluctuating light, enhanced CO_2_ utilisation, redox robustness, precursor availability, or improved compatibility with a burdensome heterologous pathway. Evolution can then be performed through serial transfer, turbidostat or chemostat cultivation while gradually increasing the relevant selective pressure. For production strains, the selection strategy should be carefully designed so that evolution does not simply enrich fast-growing non-producers; where possible, product formation should be linked to growth, biosensor output, fluorescence-activated sorting or periodic product-based screening. This is particularly important in cyanobacteria, where a mutation that reduces pathway expression may improve growth while lowering productivity. During ALE, samples should be archived at defined time points and evaluated for growth, product titre, photosynthetic performance, construct stability and stress tolerance. Whole-genome sequencing, transcriptomics and metabolomics can then be used to identify adaptive mutations, after which beneficial mutations should be reconstructed individually or in combination in a clean engineered background to confirm causality and avoid carrying forward neutral or deleterious passenger mutations. Cyanobacterial ALE has already been used to improve solvent tolerance in *Synechococcus elongatus* PCC 11801 [218] and cadmium tolerance in *Synechocystis* sp. PCC 6803 [219], while engineered hypermutation has accelerated adaptation of *Synechococcus elongatus* PCC 7942 to combined high-light and high-temperature stress [220]. These examples show that ALE, when combined with genome sequencing and reverse engineering, can reveal mutations that improve cyanobacterial robustness. For heterologous pathway engineering, the most useful future approach will be to combine ALE with rational pathway design, dynamic regulation and long-term stability screening so that evolved strains maintain both growth and production during extended cultivation.

Artificial intelligence (AI) and machine learning are also expected to play an increasingly important role in future cyanobacterial pathway engineering. AI-assisted design-build-test-learn (DBTL) workflows can integrate genome-scale metabolic models, omics datasets, enzyme information and high-throughput screening outputs to prioritise pathway variants, predict metabolic bottlenecks and reduce the number of experimental cycles required for strain optimisation [221]. In gene-expression engineering, recent deep-learning approaches have demonstrated the ability to design species-specific artificial promoters using transfer-learning models, which is particularly relevant for non-model cyanobacteria where experimentally characterised promoter datasets remain limited [156]. Similarly, pre-trained DNA models have been applied to promoter sequence design and prediction of promoter expression levels, indicating that future cyanobacterial promoter libraries could be generated and computationally refined before experimental validation [222]. AI-guided screening is also becoming increasingly relevant as high-throughput cyanobacterial platforms expand. For example, recent work combining fluorescence-activated cell sorting, deep sequencing and genetic optimisation in *Synechocystis* sp. PCC 6803 demonstrates how large variant libraries can be screened to improve enzyme expression and whole-cell biocatalytic performance [147]. Although AI-assisted cyanobacterial engineering remains at an early stage, the integration of predictive modelling, automated strain construction, high-throughput phenotyping and active-learning algorithms could substantially accelerate promoter optimisation, pathway balancing and host selection for photosynthetic bioproduction.

Another important future direction is the coupling of cyanobacterial photosynthetic carbon fixation with carbon capture, utilisation and storage (CCUS) strategies. In this context, cyanobacteria can act as biological CO_2_-conversion platforms by capturing CO_2_ through the Calvin–Benson–Bassham cycle and redirecting fixed carbon toward biomass, fuels, commodity chemicals, polymers or high-value metabolites. This links directly with carbon utilisation, because captured carbon is not only assimilated into cellular material but can also be converted into products that displace fossil-derived chemicals [223]. Recent discussions of cyanobacterial negative-emission technologies further highlight the potential of engineered cyanobacteria to contribute to carbon sequestration and bioproduction, although low productivity and cultivation cost remain major barriers to large-scale deployment [15]. Within a CCUS framework, cyanobacterial systems could be integrated with concentrated industrial CO_2_ streams, flue gas or biogenic CO_2_ sources to improve carbon supply while reducing emissions from hard-to-abate sectors. However, true carbon storage would require coupling photosynthetic capture and utilisation with durable carbon sinks, such as long-lived bioproducts, biomass-derived biochar or biocoal, mineral carbonate formation, or bioenergy with carbon capture and storage (BECCS)-type systems. Pilot-scale studies using direct flue-gas supply to algal photobioreactors also show that carbon source identity is critical, because fossil and biogenic CO_2_ inputs can produce very different life-cycle emission outcomes [224]. Therefore, future cyanobacterial biomanufacturing should be evaluated not only by product titre and productivity, but also by full carbon accounting, CO_2_ source, energy input, downstream processing and the durability of the final carbon sink. Such integration could align cyanobacterial biotechnology more closely with dual-carbon strategies aimed at carbon peaking and carbon neutrality.

Overall, future advances in cyanobacterial metabolic pathway engineering should aim to improve the functional integration of heterologous pathways within the native photosynthetic metabolism. Progress will depend on designing pathways that are not only genetically expressible but also metabolically balanced, cofactor-compatible, spatially organised, and stable during long-term cultivation. By combining pathway-level design, systems biology, dynamic regulation, cofactor engineering, and stability strategies, cyanobacteria can be developed into more reliable platforms for sustainable production of fuels, chemicals, polymers, and high-value compounds from CO_2_ and light [10].

## 7. Conclusions

Cyanobacteria represent a distinctive chassis for heterologous metabolic pathway engineering because they combine oxygenic photosynthesis, carbon fixation and biosynthetic metabolism within a single microbial system. Their ability to use light energy and CO_2_ as primary inputs provides a unique basis for developing photosynthetically driven routes to fuels, platform chemicals and high-value compounds. Over recent decades, progress in cyanobacterial transformation, chromosomal integration, promoter engineering, modular DNA assembly and CRISPR-based genome editing has expanded the capacity to introduce and regulate heterologous pathways in these hosts. The evidence reviewed in this paper indicates that successful pathway engineering in cyanobacteria depends on the functional integration of introduced biosynthetic routes with native photosynthetic and central carbon metabolism. Heterologous pathways commonly draw on metabolic precursors such as glyceraldehyde-3-phosphate, pyruvate and acetyl-CoA, as well as energy and redox cofactors including ATP, NADPH and NADH. Therefore, pathway performance is influenced by carbon flux distribution, precursor availability, cofactor regeneration and competition with native processes such as biomass formation, storage metabolism and cellular maintenance. In this context, increasing the expression of pathway enzymes alone is insufficient; efficient production requires coordinated optimisation of enzyme stoichiometry, regulatory elements, codon usage, intracellular localisation, metabolic flux and host physiological balance. Despite clear progress, several biological and engineering constraints continue to limit the productivity and scalability of cyanobacterial heterologous pathway systems. Polyploid genomes and incomplete segregation can delay the construction of stable strains, while metabolic burden and pathway-associated selective pressures may lead to loss of production phenotypes during prolonged cultivation. In addition, photosynthetic metabolism introduces constraints that are less prominent in heterotrophic production hosts, including light-dependent ATP and NADPH generation, redox fluctuations, photoinhibition, carbon allocation trade-offs and reduced volumetric productivity under dense culture conditions. These factors demonstrate that heterologous pathway performance in cyanobacteria is inseparable from the physiology of the host. Future advances should therefore focus on pathway-centred engineering strategies that connect genetic design with metabolic network function. Genome-scale modelling, flux analysis and multi-omics approaches can help identify precursor limitations, competing sinks and cofactor imbalances that restrict product formation. At the same time, tuneable promoters, RBS libraries, dynamic regulatory systems, modular pathway assembly and genome editing tools can support more precise control over pathway expression and flux distribution. Strategies that improve cofactor supply, minimise metabolic burden, enhance genetic stability and spatially organise pathway enzymes may be particularly important for complex multi-enzyme pathways. Overall, cyanobacteria offer a technically demanding platform for heterologous metabolic pathway engineering. Their future value in sustainable biotechnology will depend not only on the availability of advanced genetic tools, but also on the ability to design pathways that are metabolically compatible, cofactor-balanced, genetically stable and physiologically sustainable within photosynthetic cells. A deeper understanding of the interaction between introduced pathways and native cyanobacterial metabolism will be essential for transforming these photosynthetic organisms into reliable chassis as production platforms for biochemicals and high-value functional ingredients.

## Figures and Tables

**Table 1:** Major genetic engineering tools and molecular techniques used for the manipulation of cyanobacterial genomes.

Genetic Tool	Function	Representative Cyanobacterial Model Organisms/Strains	Advantages	Limitations	Transformation Efficiency and Scalability	Long-Term Stability and Multi-Gene Pathway Suitability	References
Natural Transformation	Uptake of extracellular DNA directly from the medium	*Synechocystis* sp. PCC 6803; *Synechococcus elongatus* PCC 7942; *Synechococcus* sp. PCC 7002	Simple DNA delivery; suitable for chromosomal integration using homologous recombination	Strain-dependent efficiency; requires natural competence	High efficiency in naturally competent strains including *Synechocystis* sp. PCC 6803 and *S. elongatus* PCC 7942; reproducible and scalable at laboratory scale without specialised equipment; not applicable to strains lacking natural competence.	Chromosomally integrated constructs stably inherited without antibiotic selection pressure; complete segregation slow in polyploid strains; limited neutral integration sites constrain multi-gene pathway construction, requiring sequential transformation rounds for complex pathways.	[85,126,127]
Conjugation	DNA transfer from E. coli donor cells to cyanobacterial recipient cells	*Anabaena* sp. PCC 7120; *Synechococcus* sp. PCC 7002; *Synechocystis* sp. CC9311	Useful for strains with low natural transformation efficiency; suitable for plasmid delivery	Multi-step process; requires donor/helper strains and compatible vectors	Moderate efficiency; reliable for strains lacking natural competence; co-cultivation with donor *E. coli* adds procedural complexity; suitable for laboratory and pilot scale.	Stability depends on integration method; plasmid delivery requires continued antibiotic selection; well suited for rapid delivery of large multi-gene constructs to otherwise inaccessible strains.	[91,128,129]
Electroporation	DNA delivery using short electrical pulses to transiently permeabilise the cell membrane	*Synechocystis* sp. PCC 6803; *Synechococcus* sp. CC9311; *Anabaena* sp. M131	Rapid physical DNA delivery without biological donor requirements; applicable to strains resistant to conjugation or with low natural competence; useful for delivering both plasmid and linear DNA constructs.	Often lower or more variable efficiency than natural transformation/conjugation; requires optimisation	Variable and often lower efficiency than natural transformation; highly protocol and strain dependent; requires optimisation of pulse parameters and growth conditions; limited scalability due to optimisation burden.	Stability of introduced constructs depends on subsequent integration method; less commonly used for stable chromosomal multi-gene insertions; useful where alternative methods fail but not preferred for complex pathway engineering.	[130,131,132]
Homologous Recombination	Stable chromosomal integration using homologous DNA flanking regions	*Synechocystis* sp. PCC 6803; *Synechococcus elongatus* PCC 7942; *Synechococcus* sp. PCC 7002; *Anabaena* sp. PCC 7120	Reliable gene insertion, deletion or replacement at defined genomic loci	Complete segregation can be slow, especially in polyploid cyanobacteria	Low frequency of individual integration events; multiple selection rounds required; slow throughput but high specificity; scalability high once stable fully segregated strains are obtained.	Produces most genetically stable strains—constructs inherited without selection pressure; complete segregation in polyploid strains is slow; limited by available neutral sites; best suited for single or small multi-gene stable insertions.	[133,134,135,136]
CRISPR-Cas9/Cas12a	RNA-guided genome editing through targeted DNA cleavage followed by repair/recombination	*Synechococcus elongatus* PCC 7942; *Synechocystis* sp. PCC 6803; *Anabaena* sp. PCC 7120	Precise targeted editing; can improve editing efficiency compared with homologous recombination alone	Cas toxicity, incomplete segregation, and off-target or lethal cleavage may occur	Moderate to high editing efficiency compared with homologous recombination; Cas12a enables simultaneous multiplex editing; Cas nuclease toxicity can reduce efficiency and cell viability in some strains.	Cas nuclease expression should be transient to avoid long-term toxicity; Cas12a better suited for multi-gene pathway engineering due to multiplexing capacity; incomplete segregation in polyploid strains must be confirmed.	[115,137,138]
CRISPR interference (CRISPRi)	Gene repression using catalytically inactive Cas protein without cutting DNA	*Synechococcus elongatus* PCC 7942; *Synechococcus* sp. PCC 7002; *Synechocystis* sp. PCC 6803	Reversible or tuneable gene repression; useful for essential genes and metabolic regulation	Requires optimisation of guide RNA design, expression level and induction conditions	Delivery efficiency similar to CRISPR-Cas9; dCas9 is non-toxic enabling stable long-term expression; scalable for dynamic metabolic regulation across diverse strains with appropriate guide RNA design.	Stable repression during continued dCas9 expression without requiring ongoing selection; reversibility useful for decoupling growth and production phases; excellent for multi-gene pathway engineering through simultaneous repression of competing pathways; repression completeness over extended cultivation should be validated.	[120,139,140]

**Table 2:** Synthesis comparison of major cyanobacterial chassis strains for heterologous metabolic pathway engineering.

Chassis Strain	Key Genetic Tools Available	Product Classes Demonstrated	Key Advantages	Major Bottlenecks	Technology Readiness Level	Key References
*Synechocystis* sp. PCC 6803 (3.57 Mb; ~2–4 chromosome copies per cell)	Natural transformation; conjugation; homologous recombination; CRISPR-Cas9/Cas12a; CRISPRi; synthetic promoter libraries (Ptrc, PpetE, PrhaBAD); RBS engineering; codon optimisation; modular DNA assembly	Ethanol; isobutanol; 3-methyl-1-butanol; 1-butanol; 1-octanol; PHB; squalene; bisabolene; limonene; β-phellandrene; taxol precursors; fatty acids; alkanes; sucrose; phycocyanin; carotenoids; antibody fragments	Most extensively characterised chassis; naturally competent (direct DNA uptake); broadest demonstrated product diversity; most developed genetic toolkit; comprehensive synthetic promoter library; extensive systems biology resources	Slow growth (doubling time 6–10 h); polyploidy delays complete construct segregation; low volumetric productivity; light attenuation limits dense cultures; photoinhibition under high light; limited tolerance to some products	TRL 3–4	[3,15,192]
*Synechococcus elongatus* PCC 7942 (2.71 Mb single chromosome; ~2–4 copies; simplest genome among model strains)	Natural transformation; homologous recombination; CRISPR-Cas9/Cas12a; CRISPRi; synthetic promoters; inducible systems (PrhaBAD); RBS and codon optimisation	Isobutanol; ethylene; 2,3-butanediol; sucrose (CscB secretion system); 1-butanol; isoprene; PHB	Naturally competent; smallest/simplest genome among classic model strains reducing competing metabolic background; well-characterised carbon fixation; closely related to fast-growing UTEX 2973 enabling direct tool transfer	Fewer characterised genetic parts and promoters than PCC 6803; limited demonstrated product diversity; polyploidy still delays segregation; less developed systems biology resources	TRL 3–4	[15,175,192]
*Synechococcus elongatus* UTEX 2973 (~2.7 Mb; >99% genome identity with PCC 7942; identified as distinct strain 2015)	Natural transformation; homologous recombination; CRISPR-Cas9 (being established); most PCC 7942 tools directly transferable; toolkit expanding rapidly	Sucrose (highest reported cyanobacterial productivity via CscB; 1.9 g/L/d); 2,3-butanediol; PHB; early terpenoid demonstrations	Fastest cyanobacterial growth rate (doubling time 1.5–1.9 h); high light and temperature tolerance; >99% genome identity with PCC 7942 permits immediate tool transfer; highest biomass productivity among model strains	Less developed genetic toolkit; limited demonstrated product diversity; requires high-intensity illumination increasing energy costs; fewer characterised promoters; limited metabolic engineering track record relative to PCC 6803 and PCC 7942	TRL 2–3	[15,208,209]
*Synechococcus* sp. PCC 7002 (3.41 Mb + 5 plasmids; ~3–5 copies; salt-adapted euryhaline genome)	Conjugation (primary delivery method); natural transformation possible; homologous recombination; CRISPR-Cas9 demonstrated; replicative and integrative plasmids available	PHB; free fatty acids; alkanes; terpenoids; hydrogen; zeaxanthin (high-value carotenoid)	Fast growth (doubling time ~2.6 h); high salinity and temperature tolerance supporting potential non-sterile outdoor cultivation; high light tolerance reducing photoinhibition; naturally elevated lipid and fatty acid content; good scalability potential	Less characterised than PCC 6803; fewer available genetic tools; relies primarily on conjugation adding complexity; limited systems biology resources; fewer demonstrated product classes; polyploidy complicates editing	TRL 2–3	[3,15,206]
*Anabaena* sp. PCC 7120 (6.41 Mb chromosome + 6 plasmids; largest genome among common model strains)	Conjugation (required; no natural competence); homologous recombination; CRISPRi demonstrated; CRISPR-Cas9 being developed; heterocyst-specific promoters (P*_petE_*, P*_nifH_*); toolkit limited relative to unicellular strains	PHB; ethanol; hydrogen (via nitrogenase); recombinant vaccine antigens (VP28); heterologous proteins; limited chemical product diversity demonstrated to date	Unique nitrogen fixation via differentiated heterocysts under N-limitation; complex multicellular organisation enabling cell-type-specific gene expression; potential co-culture partner as combined carbon and nitrogen donor; relevant for N-limited outdoor cultivation	Largest and most complex genome; difficult genetic manipulation requiring conjugation; complex regulatory and developmental networks resist metabolic redirection; slow growth; multiple plasmids complicate genetic stability; energetically costly nitrogen fixation	TRL 2–3	[3,15,210]

TRL key: TRL 2–3 (red) = experimental proof of concept in laboratory flask cultures. TRL 3–4 (amber) = validated at laboratory bioreactor scale with multiple product classes demonstrated. No cyanobacterial metabolically engineered system has yet reached pilot or industrial scale (TRL ≥5) for bulk chemical production.

## Data Availability

No new data were created or analysed in this study.
